# ﻿An island in a sea of sand: a first checklist of the herpetofauna of the Serra da Neve inselberg, southwestern Angola

**DOI:** 10.3897/zookeys.1201.120750

**Published:** 2024-05-14

**Authors:** Mariana P. Marques, Diogo Parrinha, Manuel Lopes-Lima, Arthur Tiutenko, Aaron M. Bauer, Luis M. P. Ceríaco

**Affiliations:** 1 Carnegie Museum of Natural History, 4400 Forbes Avenue, Pittsburgh PA 15213, USA Carnegie Museum of Natural History Pittsburgh United States of America; 2 CIBIO, Centro de Investigação em Biodiversidade e Recursos Genéticos, InBIO Laboratório Associado, Campus de Vairão, Universidade do Porto, 4485-661 Vairão, Portugal Universidade do Porto Vairão Portugal; 3 BIOPOLIS Program in Genomics, Biodiversity and Land Planning, CIBIO, Campus de Vairão, 4485-661, Vairão, Portugal BIOPOLIS Program in Genomics, Biodiversity and Land Planning, CIBIO Vairão Portugal; 4 Departamento de Biologia, Faculdade de Ciências da Universidade do Porto, Rua do Campo Alegre 1021, 4169-007 Porto, Portugal Departamento de Biologia, Faculdade de Ciências da Universidade do Porto Porto Portugal; 5 Friedrich-Alexander-Universität Erlangen-Nürnberg, Schlossplatz 4, 91054 Erlangen, Germany Friedrich-Alexander-Universität Erlangen-Nürnberg Erlangen Germany; 6 Department of Biology and Center for Biodiversity and Ecosystem Stewardship, Villanova University, 800 Lancaster Avenue, Villanova, PA 19085, USA Villanova University Villanova United States of America; 7 Universidade Federal do Rio de Janeiro, Museu Nacional, Departamento de Vertebrados, Quinta da Boa Vista, São Cristóvão, 20940-040 Rio de Janeiro, RJ, Brazil Universidade Federal do Rio de Janeiro Rio de Janeiro Brazil

**Keywords:** Amphibians, Angola, biodiversity, conservation, endemism, inselberg, reptiles, Southwestern Africa, taxonomy

## Abstract

The Serra da Neve inselberg in Namibe Province, southwestern Angola is the second highest peak of Angola with an elevation of 2489 m. It remains one of the least explored regions in the country, despite several endemic species having been recently described from this inselberg. Here we provide an inventory of the amphibian and reptile species ocurring in Serra da Neve and compare its fauna with that of the surrounding habitats at lower elevations. We also examine the phylogenetic affinities of the inselberg taxa. A total of 59 herpetological taxa were recorded for the Serra da Neve inselberg and its immediate surroundings. These include 11 species of amphibians, belonging to nine genera and seven different families, and 48 species of reptiles, belonging to 32 genera and 12 families. Of these, one amphibian and seven reptiles from seven different genera are strictly endemic, making the inselberg the richest region in southwestern Africa with respect to strict endemics, with one endemic reptile taxa per 127 km^2^. Not surprisingly, most of the recorded taxa belong to clades that are endemic, or at least strongly associated, with southern Africa, but two are representatives of central African clades, and another two are more closely related to eastern African highland taxa. We also provide comments on the threats to the conservation of this endemic-rich inselberg.

## ﻿Introduction

Inselbergs are isolated mountains/rock outcrops which rise more or less abruptly above a plain. Scattered across all continents, these rock outcrops are usually important biodiversity hotspots, serving as refugia for diverse plant and animal taxa (Porembski and Barthlott 2000; Burke 2005; Brand et al. 2011; Bayliss et al. 2014; Porembski et al. 2017). Acting as islands, inselbergs are usually separated and isolated from each other in a way similar to oceanic islands (MacArthur and Wilson 2001). Due to their isolation and geomorphologic delimitation from the surroundings, for some groups they represent partly independent ecosystems that are especially suitable for comparative research on the structure and dynamics of floral and faunal communities (Porembski and Barthlott 2000). Their microclimatic conditions and habitats, which usually differ from those in the surrounding lowlands, allow inselbergs to support unique biological communities and high levels of endemism (Simmons et al. 1998; Porembski and Barthlott 2000; Burke 2001, 2003, 2005; Porembski 2007; Michael et al. 2008; Brand et al. 2011, 2019; Bayliss et al. 2014). In addition, they are excellent models for studying aspects of island ecology, geographical differentiation and mechanisms influencing diversity. Due to their topography, Inselbergs are typically not used for agriculture and, except for some instances of tourism, have not been transformed by human activities. They thus constitute almost pristine habitats with special importance for conservation, and usually provide favorable conditions for flora and fauna, concerning availability of water, shade, and refuge (Porembski and Barthlott 2000).

The study of African inselbergs and other “sky-islands” has been a topic of great interest in recent years for a broad scope of scientists, from conservation biologists to systematists. This is especially true for East-African Afromontane inselberg regions, as in the case of Mount Namuli and Mount Mabu in Mozambique (e.g., Timberlake et al. 2009; Portik et al. 2013a, 2013b; Brand et al. 2011; Bayliss et al. 2014) or Mount Mulanje in Malawi (Curran et al. 2012). Due to this interest, in recent years several new endemic animal species, such as amphibians (Ceríaco et al. 2018; Conradie et al. 2018), reptiles (Branch et al. 2005; Branch and Bayliss 2009; Branch and Tolley 2010; Portik et al. 2013b; Branch et al. 2014, 2019; Marques et al. 2019, 2020, 2023a, 2023b), invertebrates (Congdon et al. 2010; Daniels and Bayliss 2012; Bilton 2014; Daniels et al. 2014, 2020), and mammals (Monadjem et al. 2010; Taylor et al. 2012; Simmons et al. 2021) have been described from inselbergs across Africa. Several studies have also provided inventories of the herpetofauna (Michael et al. 2008; Kirchhof et al. 2010; Conradie et al. 2016a; Bittencourt-Silva et al. 2020) and plant diversity (Rabarimanarivo et al. 2019; Porembski and Barthlott 2000; Burke 2003; Kandziora et al. 2022; Brand et al. 2019) of these inselbergs across the continent.

In contrast to East-African Afromontane inselberg studies, xeric southwestern African inselbergs have been largely neglected in the fauna studies (Brand et al. 2011; Elzen 1983; Griffin 2000), with most focusing only on flora and vegetation (Porembski and Barthlott 2000), and, more recently, on carnivores (Rapson et al. 2013). Northwestern Namibia and west-central Angola form an area with a considerable diversity and number of inselbergs. Surrounded by desert and savanna habitats, southwestern African inselbergs are usually rocky outcrops of diverse geology and origins that contrast with the intervening habitats (Goudie and Viles 2015). Both reptile diversity and endemism are high in rocky areas in Angola and Namibia (Bauer et al. 2023).

The landscape of southwestern Angola is characterized by the presence of isolated mountain-like rocky outcrops of subvolcanic origin mostly composed of gneisses, migmatites and granites (Pereira 1977). One of the most impressive southwestern Angolan inselbergs is Serra da Neve. Located at the northern limit of Namibe Province, southwestern Angola, and with a basal area of approximately 630 km^2^, Serra da Neve is the second highest peak of Angola, with an elevation of 2489 m (Pereira 1977). It lies in what Mendelsohn and Huntley (2023) define as “the southern escarpment landscape”, an area ranging from the Coporolo River in Benguela Province, Angola, to the Huab River in Namibia. The inselberg is covered by a Miombo forest habitat, contrasting with the surrounding lowland habitats, which are mainly dominated by Namibian woodland savanna and arid areas of Namib Desert (Grandvaux-Barbosa 1970). Although its biodiversity is still poorly known, and systematic surveys have only recently begun, the inselberg is already known to harbor an impressive number of strictly endemic species of amphibians and reptiles, such as *Poyntonophrynuspachnodes* Ceríaco, Marques, Bandeira, Agarwal, Stanley, Heinicke, Blackburn & Bauer, 2018, *Cordylusphonolithos* Marques, Ceríaco, Stanley, Bandeira, Agarwal & Bauer, 2019, *Lygodactylusbaptistai* Marques, Ceríaco, Buehler, Bandeira, Janota & Bauer, 2020, *Afroedurapraedicta* Branch, Schmitz, Lobón-Rovira, Baptista, António & Conradie, 2021, and *Acontiasmukwando* Marques, Parrinha, Tiutenko, Lopes-Lima, Bauer & Ceríaco, 2023 (Ceríaco et al. 2018; Marques et al. 2019, 2020, 2023b; Branch et al. 2021), with at least three other reptile species currently being described (MPM unpubl. data; DP unpubl. data).

Currently, no data for other taxonomic groups exist, but recent multidisciplinary surveys have also uncovered interesting and cryptic diversity within those, which will result in a better understanding of the taxonomic diversity, biogeographic patterns, and endemism of Serra da Neve. In this context, the main objective of this study is to provide a first description of Serra da Neve herpetofauna, and more specifically to 1) to provide an inventory of the occurring species as well as list taxa that have not yet been recorded, but which may be present; 2) to compare the fauna present on the inselberg with that of the surrounding lower elevation habitats; 3) to examine the phylogeographic affinities of the inselberg taxa; 4) to compare the level of endemism of Serra da Neve with other regions in Angola and southern Africa; and 5) to present a first glimpse into the major conservation threats that the inselberg herpetofauna may be facing.

## ﻿Materials and methods

**Figure 1.**
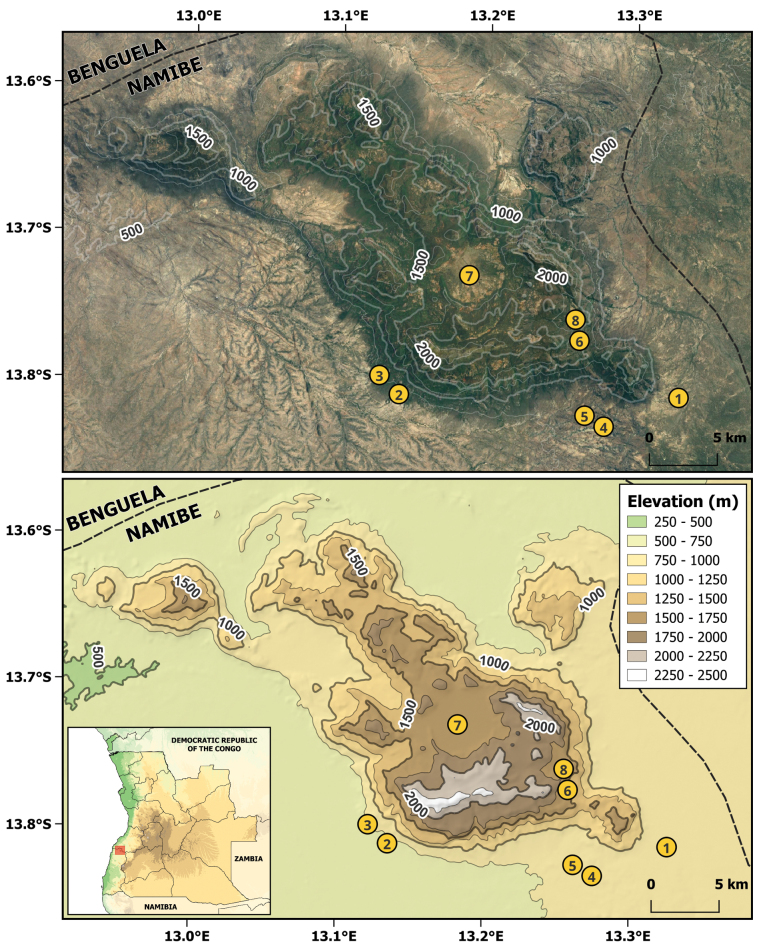
Map of the sampling localities in Serra da Neve and its surroundings; contour lines represent elevation in meters above sea level. Localities **1** road to Quilengues **2** N’Dolondolo **3** Mamué **4** Maylowe **5** 2 km N of Maylowe **6** basecamp 1 **7** Lutala Crater **8** Catchi.

Three herpetological surveys of Serra da Neve and its surrounding areas have been carried out since 2016. The first survey was conducted from 18 to 22 November 2016, the second one from 26 to 28 February 2019, and the third one from 26 October to 6 November 2022. A total of eight main sites were surveyed (Table 1, Fig. 1). The combination of sites was chosen to maximize the types of habitats surveyed, including rocky outcrops, woodlands, open grasslands, streams, and ponds, and to capture different elevations from the base to the top of Serra da Neve. Locality data are presented in decimal degrees using the WGS-84 map datum, and elevation is presented as meters above sea level.

**Table 1.** Sampling localities in and around the Serra da Neve inselberg, and respective latitude, longitude, and elevation data.

Localities	Latitude, Longitude	Elevation (m)	Map (see Fig. 1)
Road to Quilengues	-13.8159, 13.3264	587	1
N’Dolondolo	-13.8133, 13.1362	681	2
Mamué	-13.8003, 13.1229	701	3
Maylowe	-13.8355, 13.2755	798	4
2 km N of Maylowe	-13.8280, 13.2625	820	5
Basecamp 1	-13.7770, 13.2591	1488	6
Lutala Crater	-13.7325, 13.1841	1567	7
Catchi	-13.7627, 13.2564	1590	8

**Figure 2.**
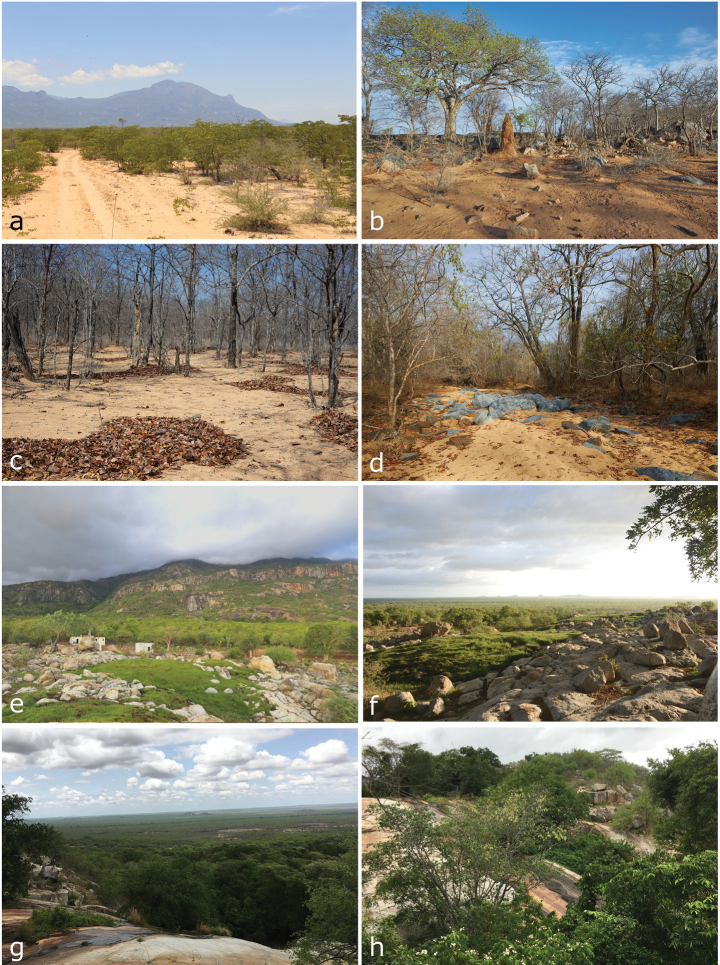
Lowland habitats in the near surroundings of the Serra da Neve inselberg **a** Mopane habitat in the vicinity of Maylowe **b** dry Mopane habitat, 2 km N of Maylowe **c** sandy areas with Mopane leaflitter near Maylowe **d** dry riverbed near Maylowe **e, f** N’Dolondolo **g, h** riparian vegetation in Mamué. Photographs by LMPC (**a**), AT (**b–d**) and IA (**e–h**).

The areas surrounding the base of Serra da Neve are all dominated by relatively dense Mopane (*Colophospermummopane*) woodlands on sandy soil (Fig. 2a–d; Huntley 2023). This habitat dominates the landscape around Maylowe village and extends for a considerable radius around Serra da Neve. The Mopane trees can be seen up to ~ 1100 m elevation on the inselberg, where they are replaced by a largely intact Miombo woodland that dominates the landscape at higher elevations. Still, at the base of Serra da Neve, some areas have interesting vegetation and geological characteristics that are worth mentioning. The N’Dolondolo area, while still dominated by Mopane woodlands, is considerably more humid than other areas near Maylowe, especially due to its sulphurous hot water spring, and its soil is less sandy, with outstanding granite outcrops present (Fig. 2e, f). Further north-west from N’Dolondolo, but still at the base of Serra da Neve, the Mamué area already presents a more complex habitat, with Mopane still present, but the landscape dominated by streams and associated riparian vegetation that extends downward from Serra da Neve, with large waterfalls (Fig. 2g, h).

**Figure 3.**
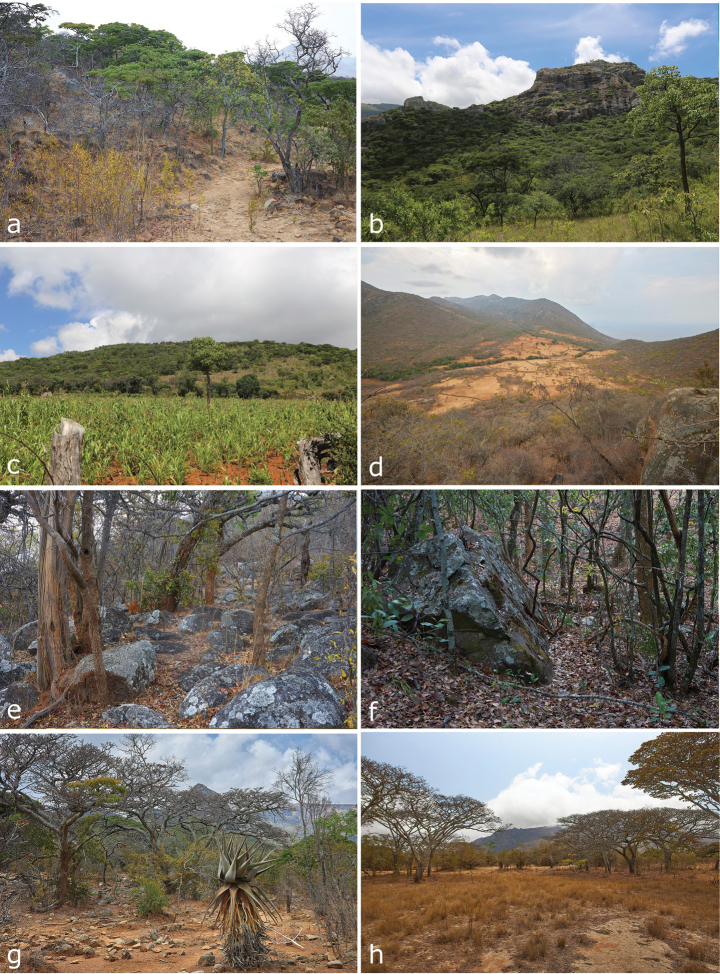
Highland habitats in Serra da Neve inselberg **a** Miombo woodland in the vicinity of Catchi **b** preserved Miombo woodlands on the way to Lutala **c** agricultural crops on the way to Lutala **d** disturbed landscape, vicinity of Catchi **e** Miombo woodland with granite outcrops, vicinity of Catchi **f** riparian vegetation on the way to Lutala crater **g, h** sparse Miombo savanna habitat at Lutala crater. Photographs by LMPC (**a–c**) and AT (**d–h**).

As elevation rises, the landscape becomes completely dominated by what Grandvaux-Barbosa (1970) described as sparse Miombo woodlands, with the presence of *Brachystegia* and *Julbernardia* trees, and the soil becomes rockier and with the conspicuous presence of large granite outcrops (Barker et al. 2015). At an elevation of ~ 1490 meters, near Basecamp 1, the area is still well-preserved, without much anthropogenic degradation. There is almost no grass, but different types of bushes (*Combretum* spp.) are present and there is a considerable accumulation of leaf-litter below the tree canopy. This type of habitat is continuous throughout the largest part of the inselberg above 900 m (pers. Obs.). The closest areas (~ 100 km) where this type of vegetation occurs are in the margins of the Escarpment around Cubal, Chongoroi and Quilengues, and then further inland in Cangandala, in the Queve and Kwanza River valleys (Grandvaux-Barbosa 1970). Catchi (1590 m), an important collecting locality in these surveys, is a small village and human impacts on the landscape are notable, as most of the plateau around the village is grazed and transformed into corn and maize plantations, or cattle pastures, with the Miombo woodlands restricted to the steeper slopes around the village. A small stream, with its respective riverine gallery, passes through the Catchi plateau, adding to the complexity of the landscape. In the main crater of the inselberg near Lutala village (1567 m), tree density is notably lower, forming an open Miombo savanna with herbaceous undergrowth, and fewer shrubs and granite outcrops in contrast to other areas of the mountain (Fig. 3).

Specimens were collected using pit fall traps, long-nooses, rubber bands, or by hand during both diurnal and nocturnal visual encounter surveys. Pitt falls were set in two different sites in a dry riverbed near Maylowe, Serra da Neve base. Each pit fall consisted of a line of four buckets, active for three days (3–5 Nov 2024). All specimens were euthanized following Villanova University animal care and use protocol #1866, preserved in 10% buffered formalin in the field, and then gradually transferred to 70% ethanol for long term storage. Liver tissues were extracted before formalin fixation and preserved in 95% ethanol. Voucher specimens were deposited in the herpetological collections of the California Academy of Sciences, USA (**CAS**); Florida Museum of Natural History, USA (**UF**); Museu Nacional de História Natural e da Ciência, Universidade de Lisboa, Portugal (**MUHNAC/MB**), Museu de História Natural e da Ciência da Universidade do Porto, Portugal (**MHNC-UP/REP**), and a subset of specimens were deposited in the reference collection of Instituto Nacional da Biodiversidade e Áreas de Conservação (**INBAC**) in Luanda, Angola. In some cases, species identifications were further confirmed by sequencing the mitochondrial 16S ribosomal RNA gene.

## ﻿Results

A total of 59 herpetological taxa were recorded for the Serra da Neve inselberg and its immediate surroundings. These include 11 species of amphibians, belonging to nine genera and seven different families, and 48 species of reptiles, belonging to 32 genera and 12 families. No crocodilians were recorded in the area. The families Scincidae and Gekkonidae were those represented by the largest number of species (14 and 10, respectively). Among the recorded species, 22 were found exclusively in the lowland areas of the inselberg base and 14 in the highlands, while 23 species were recorded throughout the study area (Table 2). Eight species are strictly endemic to the Serra da Neve inselberg, and 23 additional species are regional endemics to southwestern Angola and central and northwestern Namibia (Table 2).

**Table 2.** Synoptic table listing all recorded species, with notes on elevational distribution and endemicity. Lowland localities include Mamué, Maylowe and its surroundings, N’Dolondolo, and the road to Quilengues (below 1000 m above sea level), while highland localities refer to Catchi, Basecamp 1, and Lutala Crater (> 1000 m above sea level; see Table 1 for further details).

Taxa	Serra da Neve lowlands	Serra da Neve highlands	Strict endemic	Regional endemic (southwestern Angola and central and northwestern Namibia)
**AMPHIBIA**				
**Anura**				
**Family Pipidae**				
Genus *Xenopus*				
*Xenopuspetersii*	x	x		
**Family Bufonidae**				
Genus *Poyntonophrynus*				
*Poyntonophrynusgrandisonae*	x			x
*Poyntonophrynuspachnodes*		x	x	
Genus *Sclerophrys*				
*Sclerophryspusilla*	x	x		
**Family Microhylidae**				
Genus *Phrynomantis*				
*Phrynomantisannectens*	x			
**Family Arthroleptidae**				
Genus *Leptopelis*				
*Leptopelisanchietae*		x		x
**Family Ptychadenidae**				
Genus *Ptychadena*				
*Ptychadenaanchietae*	x			
**Family Phrynobatrachidae**				
Genus *Phrynobatrachus*				
*Phrynobatrachusnatalensis*	x	x		
**Family Pyxicephalidae**				
Genus *Amietia*				
*Amietiaangolensis*		x		
Genus *Tomopterna*				
*Tomopternaahli*	x			
*Tomopternatuberculosa*	x	x		
**REPTILIA**				
**Testudines**				
**Family Testudinidae**				
Genus *Kinixys*				
*Kinixysbelliana*	x	x		
Genus *Stigmochelys*				
*Stigmochelyspardalis*	x			
**Squamata**				
**Family Gekkonidae**				
Genus *Afroedura*				
*Afroedurapraedicta*		x	x	
Genus *Hemidactylus*				
*Hemidactylusbenguellensis*	x	x		x
Genus *Chondrodactylus*				
*Chondrodactyluspulitzerae*	x	x		x
Genus *Lygodactylus*				
*Lygodactylusbaptistai*	x	x	x	
*Lygodactylusnyaneka*	x			x
Genus *Pachydactylus*				
*Pachydactyluscaraculicus*	x			x
*Pachydactylusmaiatoi*	x	x		x
Pachydactyluscf.punctatus	x			
Genus *Rhoptropus*				
Rhoptropusaff.barnardi	x	x		x
Rhoptropusaff.montanus		x	x	
**Family Lacertidae**				
Genus *Heliobolus*				
*Helioboluscrawfordi*	x			x
Genus *Pedioplanis*				
*Pedioplanishaackei*	x			x
*Pedioplanisserodioi*	x			x
**Family Cordylidae**				
Genus *Cordylus*				
*Cordylusphonolithos*	x	x	x	
**Family Gerrhosauridae**				
Genus *Cordylosaurus*				
*Cordylosaurussubtessellatus*	x			
Genus *Gerrhosaurus*				
*Gerrhosaurus* sp.		x		
Genus *Matobosaurus*				
*Matobosaurusmaltzahni*	x	x		
**Family Scincidae**				
Genus *Acontias*				
*Acontiasmukwando*		x	x	
Genus *Mochlus*				
*Mochlussundevallii*	x			
Genus *Panaspis*				
*Panaspiscabindae*	x			
*Panaspismocamedensis*	x			
*Panaspis* sp. 1	x	x	x	
*Panaspis* sp. 2		x	x	
Genus *Sepsina*				
*Sepsinacopei*	x			x
Genus *Trachylepis*				
*Trachylepisalbopunctata*	x	x		
*Trachylepisansorgii*	x	x		x
*Trachylepisbinotata*	x			x
*Trachylepisbouri*		x		x
*Trachylepischimbana*	x	x		x
*Trachylepishuilensis*		x		x
*Trachylepislaevis*	x	x		x
**Family Chamaeleonidae**				
Genus *Chamaeleo*				
*Chamaeleodilepis*		x		
**Family Agamidae**				
Genus *Agama*				
*Agamaaculeata*	x			
*Agamaschacki*	x	x		x
**Serpentes**				
**Family Typhlopidae**				
Genus *Afrotyphlops*				
*Afrotyphlopsschlegelipetersii*	x			
**Family Leptotyphlopidae**				
Genus *Leptotyphlops*				
Leptotyphlopscf.scutifrons		x		
**Family Pythonidae**				
Genus *Python*				
*Pythonnatalensis*	x			
**Family Viperidae**				
Genus *Bitis*				
(Subgenus Macrocerastes)				
Bitis (Macrocerastes) gabonica		x		
Genus *Causus*				
*Caususnasalis*	x			x
**Family Lamprophiidae**				
Genus *Boaedon*				
*Boaedonvariegatus*	x	x		x
Genus *Hemirhagerrhis*				
*Hemirhagerrhisviperina*	x	x		x
Genus *Lycophidion*				
*Lycophidionhellmichi*	x	x		x
Genus *Psammophis*				
*Psammophissubtaeniatus*	x	x		
*Psammophylaxtritaeniatus*		x		
**Family Colubridae**				
Genus *Dasypeltis*				
*Dasypeltisscabra*	x			
Genus *Telescopus*				
*Telescopussemiannulatuspolystictus*	x			

### ﻿Endemicity levels

Serra da Neve currently harbors a total of eight strictly endemic herpetological species (one amphibian and seven reptiles, from seven different genera). We consider strictly endemic taxa to be those currently only known to occur within the area defined by the base of the Serra da Neve inselberg (see Fig. 1). These include one species of bufonid frog *Poyntonophrynuspachnodes* (family Bufonidae), one cordylid lizard, *Cordylusphonolithos* (family Cordylidae), three geckos, *Lygodactylusbaptistai*, *Afroedurapraedicta*, and Rhoptropusaff.montanus (family Gekkonidae), and three skinks, *Acontiasmukwando*, *Panaspis* sp. 1, and *Panaspis* sp. 2 (family Scincidae). No strictly endemic snakes or chelonians are known from Serra da Neve. Besides the strictly endemic taxa, the inselberg hosts a number of other regional highland endemics, such as *Leptopelisanchietae* (Bocage, 1873), *Trachylepishuilensis* (Laurent, 1964), *Trachylepisansorgii* (Boulenger, 1907), *Trachylepisbouri* Ceríaco, Marques, Parrinha, Tiutenko, Weinell, Butler & Bauer, 2024, and *Pachydactylusmaiatoi* Marques, Parrinha, Ceríaco, Brennan, Heinicke & Bauer, 2023, all associated with the highland areas of southwestern Angola.

When numbers of strictly endemic taxa on Serra da Neve and the other southwestern African highlands are compared, the inselberg stands amongst the richest in the region (Table 3; Bauer et al. 2023; Becker et al. 2023). Serra da Neve hosts a total of seven strictly endemic reptile species, the highest level of endemicity in the country. The Huíla Escarpment and Plateau in southwestern Angola (with an approximate area of 18000 km^2^), harbor only five strictly endemic reptile species (Table 3; Bauer et al. 2023; Becker et al. 2023). All other inselbergs, both in Angola and Namibia, have a maximum of two strictly endemic species (Table 3; Bauer et al. 2023; Becker et al. 2023). Serra da Neve’s strictly endemic numbers are even more striking when considering its area. With an approximate area of 630 km^2^, Serra da Neve is undoubtedly the richest region in southwestern Africa with respect to strict endemics per unit of area, with one endemic reptile taxa per 127 km^2^ (Table 3; Bauer et al. 2023), and much more similar to or higher than those found on endemic-rich oceanic islands (Ceríaco et al. 2022; Bauer et al. 2022), or South American table mountains, known as “tepuis” (Recoder et al. 2020; Fouquet et al. 2023).

**Table 3.** Comparison of herpetofaunal endemicity levels between Angolan and Namibian inselbergs. Data from Bauer et al. (2023) and Becker et al. (2023).

Country	Inselberg / Highlands	Strictly endemic species (Amphibians / Reptiles)	Strictly highland but not strictly endemic (Amphibians / Reptiles)
**Namibia**	Brandberg	– / –	– / 2
	Erongo Mts	– / –	– / 1
	Spitzkoppe	– / –	– / 1
	Swakop-Kahn inselbergs	– / –	– / 3
	Central Highlands	– / 1	– / 4
	Tiras Mountains	– / 1	– / 3
	Huns-Orange Mts	– / –	– / 3
	Baynes-Otjihipa	– / 1	– / –
	Entedeka Mts	– / –	– / –
	Karasberg	– / 1	– / 3
	Klein Karasberg	– / –	– / 1
	Nubib Mts	– / 1	– / 2
	Aus Mts	–	– / 1
	Otavi Highlands	– / 2	– / –
	Huab Outliers	– / –	– / 1
	Brukkaros	– / –	– / –
	Onder-Rooirand	– / –	– / 1
	Interior Plateau	– / 1	– / –
	Namuskluft Mts	– / –	1 / 1
	south Otjihipa Mts	– / –	– / –
	Waterberg Plateau	– / 2	– / –
	Skerpioenkop	– / –	– / 1
	Central Group south to Karasberg exclusive of desert inselbergs	– / –	– / 1
	Naukluft Mts	– / –	– / 1
	Tsaris Mts	– / –	– / 1
	Gamsberg	– / –	– / 1
	Rantberge	– / –	– / 1
	Central Plateau	– / –	– / 1
	Rooikoppe	– / –	– / 1
	Auas Mountains	– / –	– / 1
**Angola**	Namba	1 / 1	/ –
	Serra da Neve	1 / 7	1 / 4
	Huíla Escarpment and Plateau	2 / 5	1 / 4
	Central Plateau	2 / 1	1 / 6
	Pungo Andongo	– / 1	– / –
	Mt. Moco	– / 1	– / 1
	Mombolo	– / 1	
	Congulu Escarpments	3 / –	2

### ﻿Biogeographic affinities

Not surprisingly, most of the recorded taxa belong to clades endemic to, or at least strongly associated, with southern Africa. The exceptions are Bitis (Macrocerastes) gabonica (Duméril, Bibron & Duméril, 1854), *Panaspiscabindae* (Bocage, 1866) *Acontiasmukwando*, and *Lygodactylusbaptistai*, of which the first two are representatives of central African clades, and the latter two are more closely related to eastern African highland taxa. The subgenus Macrocerastes, of which large-bodied vipers such as *Bitisnasicornis* (Shaw, 1792), *B.rhinoceros* (Schlegel, 1855), *B.parviocula* (Böhme, 1977) and *B.gabonica* are members, is a group predominantly associated with central African habitats (Barlow et al. 2019; Ceríaco et al. 2020a), even if some of these species have some populations in southern (*B.gabonica*) and eastern Africa (*B.parviocula*). Within this group, is *B.heraldica* (Bocage, 1889), a species endemic to the Angolan central highlands, and the only member of the subgenus which is small-bodied (Ceríaco et al. 2020a). The specimen of *B.gabonica* from Serra da Neve represents the southwestern-most record of the subgenus in the continent.

A similar distribution pattern can also be observed for *Panaspiscabindae* and the putative new species *Panaspis* sp. 1 (MPM unpubl. data). Although the genus *Panaspis* is relatively diverse and widely distributed in southern Africa (Medina et al. 2016), the distribution of *P.cabindae* ranges from southwestern Republic of the Congo and the Democratic Republic of the Congo southwards to central and southwestern Angola through woodlands associated with the Angolan Escarpment (Ceríaco et al. 2020b). The putative new species *Panaspis* sp. 1 belongs to the same Central African lineages as *P.cabindae*, the same clade as the species from the Gulf of Guinea Oceanic islands (Ceríaco et al. 2020b; MPM unpubl. data).

On the other hand, *Lygodactylusbaptistai* belongs to a lineage comprising East African species (Marques et al. 2020). As part of the *A.occidentalis* species complex, a group whose distribution covers most of southern Africa, *A.mukwando* is more closely related to *A.percivali*, a species endemic to the Eastern Arc Mountains of northeastern Tanzania and southeast Kenya, than to other lineages of the complex occurring in Namibia and Angola (Marques et al. 2023b). This pattern is also present in the new species *Panaspis* sp. 2, currently under description (MPM unpubl. data). Among the taxa with southern African affinities there are particularly arid-adapted lizards, which are mostly restricted to the dry lowlands of the inselberg, such as *Pedioplanishaackei* Conradie, Measey, Branch & Tolley, 2012, *Pedioplanisserodioi* Parrinha, Marques, Heinicke, Khalid, Parker, Tolley, Childers, Conradie, Bauer & Ceríaco, 2021, *Helioboluscrawfordi* Marques, Ceríaco, Heinicke, Chehouri, Conradie, Tolley & Bauer, 2022, and *Pachydactyluscaraculicus* Fitzsimons, 1959. On the other hand, the more mesic highlands of the inselberg also support taxa that are typically associated with the highlands of the Escarpment and the Central Plateau, such as *Trachylepisansorgii*, *Trachylepishuilensis*, *Psammophylaxtritaeniatus* (Günther, 1868), and *Leptopelisanchietae*.

### ﻿Taxonomic accounts

**Amphibia, Anura**

#### ﻿Family Pipidae Gray, 1825

**Genus *Xenopus* Wagler, 1827**

##### ﻿ Xenopus petersii

Taxon classificationAnimaliaAnuraPipidae

Bocage, 1895

B2E3DBD5-4955-5777-9293-FB8BC477B48C

Fig. 4a, b

###### Records.

Catchi, small stream near basecamp [-13.7630, 13.2513, 1595 m] (MUNHAC/MB04-001066); Maylowe, inside a well [-13.8349, 13.2765, 803 m] (MUNHAC/MB04-001067−001091).

**Figure 4.**
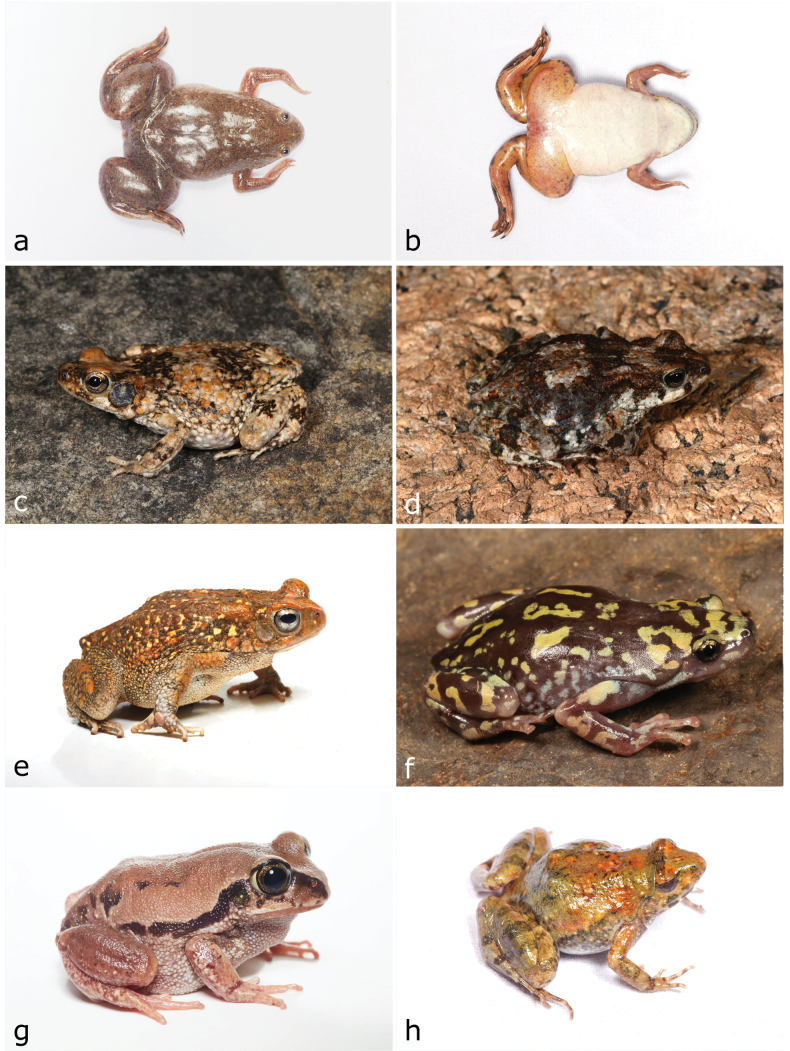
**a, b***Xenopuspetersii***c***Poyntonophrynusdombensis***d***Poyntonophrynuspachnodes***e***Sclerophryspusilla***f***Phrynomantisannectens***g***Leptopelisanchietae***h***Phrynobatrachusnatalensis*. Photographs by AT (**a, b, e, g, h**); LMPC (**c, f**) and IA (**d**).

###### Comments.

Traditionally considered as a subspecies of *Xenopuslaevis* (Daudin, 1802), Furman et al. (2015) reclassified all central and western Angolan populations as *X.petersii*. Frétey et al. (2018) designated as lectotype of the species a specimen collected by José d’Anchieta in Benguela Province, Angola. This species is known from several localities in southwestern regions of the country (see Marques et al. 2018) and the Serra da Neve population is within the expected distribution range of *X.petersii* in the province.

#### ﻿Family Bufonidae Gray, 1825

**Genus *Poyntonophrynus* Frost, Grant, Faivovich, Bain, Haas, Haddad, de Sá, Channing, Wilkinson, Donnellan, Raxworthy, Campbell, Blotto, Moler, Drewes, Nussbaum, Lynch, Green & Wheeler, 2006**

##### ﻿ Poyntonophrynus grandisonae

Taxon classificationAnimaliaAnuraBufonidae

(Poynton & Haacke, 1993)

CD569C8F-145A-5C6A-A22D-77C06503794C

Fig. 4c

###### Records.

N’Dolondolo [-13.8133, 13.1362, 889 m] (CAS 262731, 262732; UF 184185–184187; INBAC/AMB 10337, 10339).

###### Comments.

These are the first specimens of *P.grandisonae* collected since the original description by Poynton and Haacke (1993) and used by Ceríaco et al. (2018) to provide the first molecular data for *P.grandisonae*. The natural history and distribution of this species remain poorly known. The species is endemic to Angola and restricted to the southwestern regions of Namibe Province. It is associated with xeric vegetation in low-elevation areas (Marques et al. 2018; Baptista et al. 2023).

##### ﻿ Poyntonophrynus pachnodes

Taxon classificationAnimaliaAnuraBufonidae

Ceríaco, Marques, Bandeira, Agarwal, Stanley, Bauer, Heinicke & Blackburn, 2018

BE017F6C-6D5E-561B-8D7D-9D5D84281855

Fig. 4d

###### Records.

Basecamp 1 [-13.7770, 13.259, 1488 m] (UF 184183, 184184, 190278, 190279; CAS 262730, INBAC/AMB 10209; INBAC/LMPC 1262–1264; MUHNAC/MB04-000999, 001092).

###### Comments.

This recently described species is only known from Serra da Neve and considered a strict endemic (Ceríaco et al. 2018; Baptista et al. 2023). Additional material for this species was collected from the type locality by the second expedition to the inselberg in 2019. This highland specialist was found in an area with moist soil under leaf-litter and rocks, at elevations greater than 1000 m. Recent research by Baptista et al. (2023) provided new data regarding the phylogenetic relationship of this Serra da Neve endemic, indicating that it is closely related to the arid-adapted *P.dombensis* and *P.damaranus*.

####### Genus *Sclerophrys* Tschudi, 1838

##### ﻿ Sclerophrys pusilla

Taxon classificationAnimaliaAnuraBufonidae

(Mertens, 1937)

BF75E64E-8767-5085-BCA5-F22DDC4FB4BF

Fig. 4e

###### Records.

Basecamp 1 [-13.7770, 13.2591, 1488 m] (CAS 263036, 263037; INBAC/AMB 10206); vic. N’Dolondolo [-13.8087, 13.1352, 731 m] (CAS 263025, 263030); N’Dolondolo [-13.8133, 13.1362, 593 m] (UF 187174); Maylowe [-13.8342, 13.2767, 803 m] (UF 190289; MUNHAC/MB04-001093); 2 km N of Maylowe [-13.8280, 13.2625, 820 m] (MUNHAC/MB04-001094).

###### Comments.

The species has a wide distributional range in central and southern Angola (Marques et al. 2018). The Angolan population was for some time treated as *Sclerophrysmaculata* (Hallowell, 1854), until Poynton et al. (2016) established that *S.maculata* is restricted to West Africa, while *S.pusilla* is mainly distributed across eastern and southern Africa, including Angola. This species is common in Serra da Neve and its surroundings. It is usually found near leaf litter.

#### ﻿Family Microhylidae Günther, 1858

**Genus *Phrynomantis* Peters, 1867**

##### ﻿ Phrynomantis annectens

Taxon classificationAnimaliaAnuraMicrohylidae

Werner, 1910

667A0A6B-96E1-5FFB-8DBA-EBF4D94F3E85

Fig. 4f

###### Records.

N’Dolondolo [-13.8004, 13.1362, 897 m] (UF 187250, 187251; INBAC/AMB 10344); Maylowe [-13.8342, 13.2767, 803 m] (UF 190275).

###### Comments.

Endemic to southwestern Africa from southwestern Angola southwards through Namibia to the arid regions of northern South Africa (Marques et al. 2018; Channing and Rödel 2019). In Angola, *P.annectens* is restricted to the coastal lowlands of the country (Marques et al. 2018). The species is frequently associated with inselbergs and other rocky outcrops in arid regions (Channing 2001; Channing and Rödel 2019). Our specimens were found inside crevices at the base of Serra da Neve. Ceríaco et al. (2021) discussed the taxonomic and nomenclatural history of *P.annectens* and provided an additional record for the country.

#### ﻿Family Arthroleptidae Mivart, 1869

**Genus *Leptopelis* Günther, 1859**

##### ﻿ Leptopelis anchietae

Taxon classificationAnimaliaAnuraArthroleptidae

(Bocage, 1873)

C16F34DE-0864-52AC-A043-96DC8D7C4BE3

Fig. 4g

###### Records.

Catchi, near small stream [-13.7630, 13.2514, 1595 m] (MUNHAC/MB04- 001095, 001096).

###### Comments

. An Angolan endemic, distributed throughout much of the western half of the country (Marques et al. 2018; Becker et al. 2023). The collected specimens were heard croaking from a small bush at night, a common event especially after rain. Considered a cryptic species complex by several authors (Perret 1976; Schiøtz 1999). Becker et al. (2023) did not record the species in Serra da Neve.

#### ﻿Family Ptychadenidae Dubois, 1987

**Genus *Ptychadena* Boulenger, 1917**

##### ﻿ Ptychadena anchietae

Taxon classificationAnimaliaAnura Ptychadenidae

(Bocage, 1867)

326F7D99-4BB5-505E-86AB-7E428FB2748D

###### Records.

N’Dolondolo [-13.8004, 13.1362, 897 m] (CAS 263024; UF 187280; INBAC/AMB 10308).

###### Comments.

The species is broadly distributed from western to eastern Angola and is widespread extralimitally (Marques et al. 2018; Channing and Rödel 2019), typically associated with savanna and grassland habitats.

#### ﻿Family Phrynobatrachidae Laurent, 1941

**Genus *Phrynobatrachus* Günther, 1862**

##### ﻿ Phrynobatrachus natalensis

Taxon classificationAnimaliaAnuraPhrynobatrachidae

(Smith, 1849)

B6DC4AE1-B832-5359-A967-5BF4D80EEE8A

Figs 4e , 5a

###### Records.

Mamué riparian area [-13.8004, 13.1246, 732 m] (UF 187249); Catchi, small stream near basecamp [-13.7630, 13.2513, 1595 m] (MUNHAC/MB04-001097–001146).

**Figure 5.**
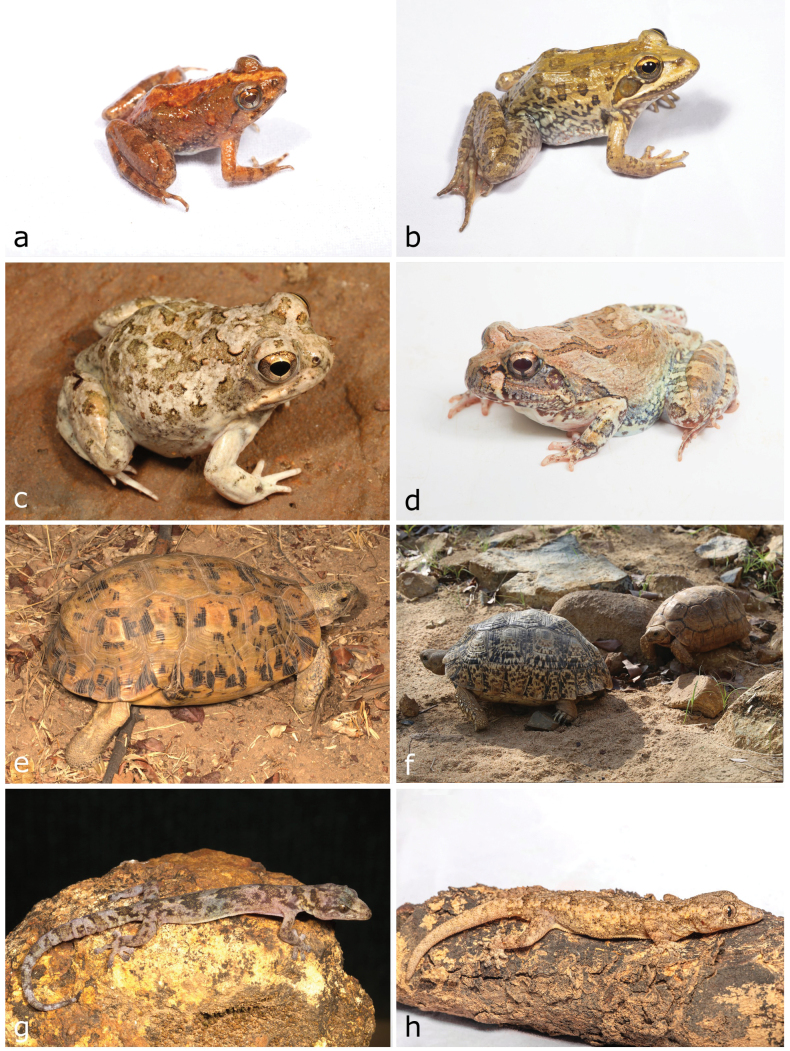
**a***Phrynobatrachusnatalensis***b***Amietiaangolensis***c***Tomopternaahli***d***Tomopternatuberculosa***e***Kinixysbelliana***f***Stigmochelyspardalis***g***Afroedurapraedicta***h***Hemidactylusbenguellensis*. Photographs by AT (**a, b, d, h**) and LMPC (**c, e–g**).

###### Comments.

The species, as currently understood, is likely to contain multiple undescribed cryptic species across its large distribution in the savanna and grassland regions of sub-Saharan Africa (Zimkus et al. 2010). Based on newly generated molecular data (LMPC unpub. data), we conclude that the population from Serra da Neve belongs to the same lineage as topotypical populations in South Africa. The specimens were collected on the margins of small streams, both in the lowlands and highlands of the inselberg.

#### ﻿Family Pyxicephalidae Bonaparte, 1850

**Genus *Amietia* Dubois, 1987**

##### ﻿ Amietia angolensis

Taxon classificationAnimaliaAnura Pyxicephalidae

(Bocage, 1866)

E90CF60C-82DE-52AF-8658-36E1BCA52D4B

Fig. 5b

###### Records.

Catchi, small stream near basecamp [-13.7630, 13.2513, 1595 m] (MUNHAC/MB04-001147−001154).

###### Comments.

Formerly considered a widespread species in western and central Africa, is now restricted to Angola (Channing and Baptista 2013; Channing et al. 2016). The species was frequently encountered in the rocky margins of small streams and ponds at the top of the inselberg.

####### Genus *Tomopterna* Duméril & Bibron, 1841

##### ﻿ Tomopterna ahli

Taxon classificationAnimaliaAnuraPyxicephalidae

(Deckert, 1938)

E1ACC362-07E7-5DF0-B93A-E114B414E1E7

Fig. 5c

###### Record.

Maylowe [-13.8342, 13.2767, 803 m] (UF 190305).

###### Comments.

Ceríaco et al. (2016) provided the first record of this species for Angola as *Tomopternadamarensis* Dawood & Channing, 2002. Heinicke et al. (2017a) used morphological and mtDNA data to show that this species is broadly distributed in Angola and Namibia and suggested that some records of *Tomopterna* from southern Angola could correspond to this species. Channing and Becker (2019) showed that *Tomopternaahli* was a senior synonym of *T.damarensis*. All Angolan specimens previously assigned to *T.damarensis* (Ceríaco et al. 2016, Heinicke et al. 2017a; Marques et al. 2018) should thus be referred to as *T.ahli*.

##### ﻿ Tomopterna tuberculosa

Taxon classificationAnimaliaAnuraPyxicephalidae

(Boulenger, 1882)

6A3B4231-B155-5605-902B-BD12A6D4395A

Fig. 5d

###### Records.

Basecamp 1 [-13.7770, 13.2591, 1488 m] (CAS 263038, 263039); Mamwé riparian area [-13.8006, 13.1230, 706 m] (UF 187293); N’Dolondolo [-13.8133, 13.1362, 681 m] (UF 187294); Maylowe [-13.8342, 13.2767, 803 m] (MUNHAC/MB04-001155); Catchi, basecamp [-13.7627, 13.2562, 1597 m] (MUNHAC/MB04-0011556).

###### Comments.

The species has a wide distribution in western Angola (Marques et al. 2018). These specimens represent the first record of the species in Namibe Province since those that were reported by Bocage (1895). *Tomopternatuberculosa* appears to occur in sympatry with *T.ahli* at Serra da Neve and across the southwestern areas of Namibe Province.

#### ﻿Reptilia, Testudines

**Family Testudinidae Batsch, 1788**

##### Genus *Kinixys* Bell, 1827

###### ﻿ Kinixys belliana

Taxon classificationAnimaliaTestudinesTestudinidae

Gray, 1863

B20D1573-8556-55A5-A65A-0F6331ED9A4B

Fig. 5e

####### Records.

Catchi surroundings [-13.7577, 13.2543, 1576 m] (MUNHAC/MB03-001548, 001549 only tissue); 2 km N of Maylowe [-13.8280, 13.2625, 820 m] (MUNHAC/MB03-001550, only tissue).

####### Comments.

A wide-ranging species from eastern Africa to southwestern and central Angola. Historically, the taxonomic status of *Kinixys* populations in Angola has been uncertain (for further discussion see Marques et al. 2018), although according to the most recent sub-Saharan Africa chelonian phylogeny (Fritz et al. 2022), the Angolan material should be assigned to *Kinixysbelliana*. This species is frequently consumed a delicacy by the Mucubal tribe at the base of Serra da Neve (pers. obs.).

##### Genus *Stigmochelys* Gray, 1873

###### ﻿ Stigmochelys pardalis

Taxon classificationAnimaliaTestudinesTestudinidae

(Bell, 1828)

7DD3AE52-9A5F-5B33-8C56-A9CBF3EAE057

Fig. 5f

####### Record.

2 km N of Maylowe [-13.8280, 13.2625, 820 m] (MUNHAC/MB03-001551, only tissue).

####### Comments.

*Stigmochelyspardalis* is a large-bodied species occurring from northern Somalia southwards through eastern Africa to South Africa, and westwards to Namibia and Angola (Marques et al. 2018). The species has been recorded in the southern provinces of the country (Marques et al. 2018).

#### ﻿Reptilia, Squamata

**Family Gekkonidae Gray, 1825**

**Genus *Afroedura* Loveridge, 1944**

##### ﻿ Afroedura praedicta

Taxon classificationAnimaliaSquamataGekkonidae

Branch, Schmitz, Lobón-Rovira, Baptista, António & Conradie, 2021

BE4AF6D1-7693-50A1-9D22-5EDC6BC62B1C

Fig. 5g

###### Records.

Catchi, rock outcrops near basecamp [-13.7653, 13.2571, 1645 m] (MUNHAC/ MB03-001552−001554).

###### Comments.

This recently described species is only known from the Serra da Neve inselberg and is considered a strict endemic (Branch et al. 2021; Conradie et al. 2023). This highland specialist was found in vertical walls of large granite boulders at night. A juvenile (MUNHAC/ MB03-001552) was collected in rock crevices near a communal laying site with dozens of hatched eggs.

####### Genus *Hemidactylus* Goldfuss, 1820

##### ﻿ Hemidactylus benguellensis

Taxon classificationAnimaliaSquamataGekkonidae

Bocage, 1893

8D4FECA3-5204-5DCC-9EA5-AB3F8ED5AA8F

Fig. 5h

###### Records.

Basecamp 1 [-13.7770, 13.2591, 1488 m] (CAS 263367–263372); N’Dolondolo [-13.8133, 13.1362, 681 m] (CAS 263536–263540; UF 187202; INBAC/AMB 10237, 10245); vic. N’Dolondolo [-13.8068, 13.1351, 754 m] (CAS 263549); Maylowe [-13.8342, 13.2767, 803 m] (CAS 266144); Catchi, rock outcrops near basecamp [-13.7653, 13.2571, 1645 m] (MUNHAC/MB03-001555, 001556); MPLA post near Catchi [-13.7618, 13.2514, 1614m] (MUNHAC/MB03-001799); 2 km N of Maylowe [-13.8280, 13.2625, 820 m] (MUNHAC/MB03-001557, 001558).

###### Comments.

The species is endemic to southwestern Angola and northern Namibia (Marques et al. 2018; Ceríaco et al. 2020c; Lobón-Rovira et al. 2021). Although *H.benguellensis* appears to be very common in the Angolan Escarpment areas, it has also been found in more coastal environments in southwestern Namibe Province (Lobón-Rovira et al. 2021). The species is strongly associated with rupiculous habitats.

####### Genus *Chondrodactylus* Peters, 1870

##### ﻿ Chondrodactylus pulitzerae

Taxon classificationAnimaliaSquamataGekkonidae

(Schmidt, 1933)

73B6DC18-30B6-5C2B-B6D6-371CBCAEEFC9

Fig. 6a

###### Records.

Basecamp 1 [-13.7770, 13.2591, 1488 m] (CAS 266367–CAS 266369, 266371; INBAC/AMB 10200); N’Dolondolo [-13.8133, 13.1362, 681 m] (CAS 266370); Maylowe [-13.8342, 13.2767, 803 m a.s.l] (CAS 266114); Catchi, rock outcrops near basecamp [-13.7653, 13.2571, 1645 m] (MUNHAC/MB03-001559− 001565); 2 km N of Maylowe [-13.8280, 13.2625, 820 m] (MUNHAC/MB03-001566−001568).

**Figure 6.**
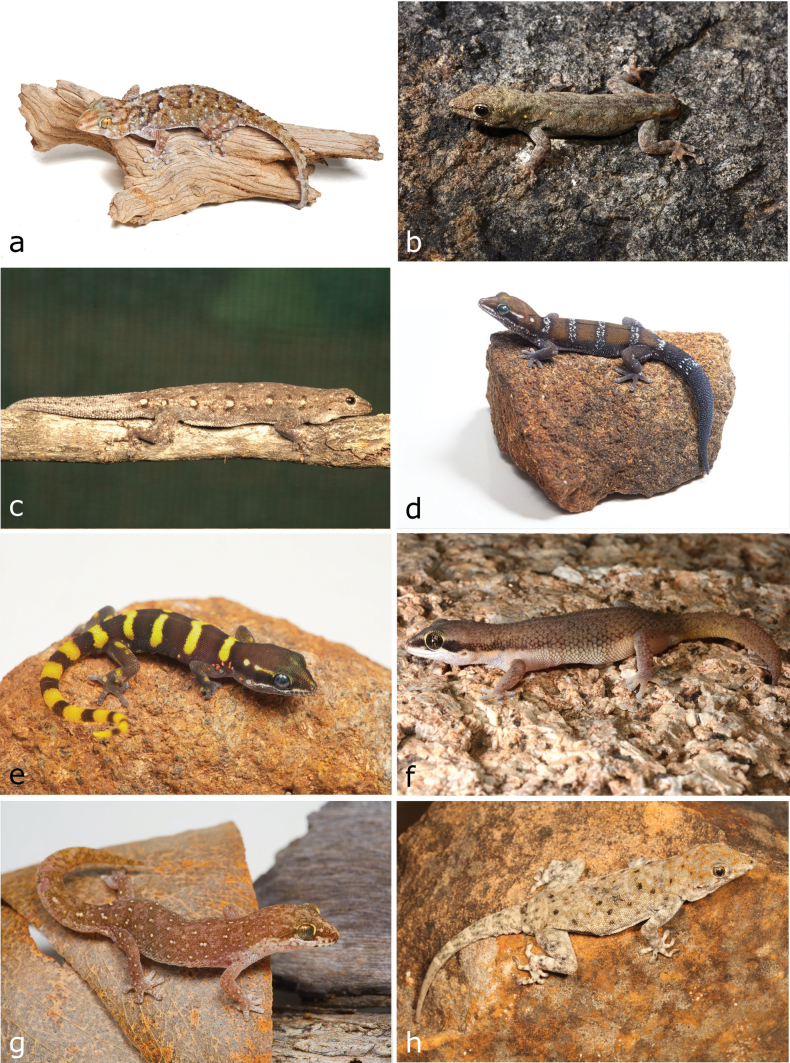
**a***Chondrodactyluspulitzerae***b***Lygodactylusbaptistai***c***Lygodactylusnyaneka***d, e***Pachydactyluscaraculicus* (adult and juvenile) **f***Pachydactylusmaiatoi***g**Pachydactyluscf.punctatus**h**Rhoptropusaff.barnardi. Photographs by AT (**a, d, e, g**); IA (**b, f**) and LMPC (**c, h**).

###### Comments.

The genus has recently been reviewed by Heinz et al. (2021). The species is quite widespread in Angola (Marques et al. 2018; Heinz et al. 2021), and it occurs at different elevations on Serra da Neve and its surrounding areas.

####### Genus *Lygodactylus* Gray, 1864

##### ﻿ Lygodactylus baptistai

Taxon classificationAnimaliaSquamataGekkonidae

Marques, Ceríaco, Buehler, Bandeira, Janota & Bauer, 2020

B7C84791-AE9E-5EF9-B72E-CF528B3F4E54

Fig. 6b

###### Records.

Mamué riparian area [-13.8008, 13.1235, 715 m] (CAS 263557), [-13.8004, 13.1246, 748 m] (CAS 263551); MPLA post near Catchi [-13.7618, 13.2514, 1614 m] (MUNHAC/MB03-001569−001571); near Ondjili, between Catchi and Lutala crater (MUNHAC/MB03-001572).

###### Comments.

This recently described species is only known from the Serra da Neve inselberg and is considered a strict endemic. The species was recently described from the inselberg by Marques et al. (2020). *Lygodactylusbaptistai* appears to be the single representative of this lineage in southwestern Africa. Morphologically it is also more similar to those species found on inselbergs of Mozambique such as *L.rex* Broadley, 1963 and *L.regulus* Portik, Travers, Bauer & Branch, 2013 (Portik et al. 2013b) than to the other species known from Angola, *L.angolensis* Bocage, 1896 and *L.nyaneka* Marques, Ceríaco, Buehler, Bandeira, Janota & Bauer, 2020, both restricted to Miombo forested areas, or *L.lawrencei* Hewitt, 1926, an arid zone specialist.

##### ﻿ Lygodactylus nyaneka

Taxon classificationAnimaliaSquamataGekkonidae

Marques, Ceríaco, Buehler, Bandeira, Janota & Bauer, 2020

4228BA81-31C1-554D-92D2-CCC999A492AD

Fig. 6c

###### Record.

Mamué riparian area [-13.8008, 13.1235, 715 m] (CAS 263556).

###### Comments.

A recently described species from the central and southwestern regions of the country and neighboring northern Namibia, with records from Epupa Falls (Marques et al. 2020). The individual collected from Serra da Neve occurs in sympatry with the strictly endemic *L.baptistai*.

####### Genus *Pachydactylus* Wiegmann, 1834

##### ﻿ Pachydactylus caraculicus

Taxon classificationAnimaliaSquamataGekkonidae

FitzSimons, 1959

05B4931D-24F9-51C0-84CE-DB0BCB042AA0

Fig. 6d, e

###### Records.

N’Dolondolo [-13.8133, 13.1362, 681 m] (CAS 10283, 10347); Maylowe [-13.8342, 13.2767, 803 m] (CAS 266145; MUNHAC/MB03-001573); 2 km N of Maylowe [-13.8280, 13.2616, 804 m] (MUNHAC/MB03-001574); 2 km N of Maylowe, rock outcrops near basecamp [-13.8280, 13.2646, 8020 m] (MUNHAC/MB03-001575−001581).

###### Comments.

The species is known from southwestern Angola and northwestern Namibia (Marques et al. 2018). It is part of a diverse and primarily rupiculous “northwestern clade” of *Pachydactylus* (Bauer and Lamb 2005; Heinicke et al. 2011), sister to *P.angolensis* Loveridge, 1944 and *P.maiatoi* (Heinicke et al. 2017b; Marques et al. 2023a). Usually found in crevices and cracks of granitic boulders.

##### ﻿ Pachydactylus maiatoi

Taxon classificationAnimaliaSquamataGekkonidae

Marques, Parrinha, Ceríaco, Brennan, Heinicke & Bauer, 2023

A7669B76-369F-5814-AEB7-01B7CB1F5E8D

Fig. 6f

###### Records.

N’Dolondolo [-13.8133, 13.1362, 681 m] (CAS 266484, 266485); Basecamp 1 [-13.7770, 13.2591, 1488 m] (CAS 266486); Maylowe [-13.8355, 13.2755, 798 m] (MUNHAC/MB03-001246); Catchi, rock outcrops near basecamp [-13.7653, 13.2571, 1645 m] (MUNHAC/MB03-001247); 2 km N of Maylowe [-13.8289, 13.2625, 820 m] (MUNHAC/MB03-001248).

###### Comments.

Heinicke et al. (2017b) identified putative species-level diversity within the *Pachydactylusangolensis* group. Based on a combination of morphological, meristic and DNA sequence data, it was possible to separate two different taxa, a “coastal” and “inland” form of *P.angolensis* group. Marques et al. (2023a) described the inland form as *P.maiatoi*. The recently described species appears to be restricted to southwestern Angola, namely in the inland regions of Namibe Province and along the highlands associated with the Escarpment in Huíla Province. This species is usually found under rocks in areas with some vegetation, in highland regions.

##### ﻿ Pachydactylus cf. punctatus

Taxon classificationAnimaliaSquamataGekkonidae

Peters, 1854

57207C6D-2CD0-51D6-BB8B-316B59EB7EE5

Fig. 6g

###### Records.

2 km N of Maylowe [-13.8289, 13.2625, 820 m] (MUNHAC/MB03-001582− 001591).

###### Comments.

*Pachydactyluspunctatus* is a widespread species complex extending from South Africa northwards to Malawi, the former Katanga Province of the Democratic Republic of the Congo and southern Angola (Marques et al. 2018). In Angola itself, Heinz (2011) noted the presence of four species-level divergent lineages. As the taxonomy and nomenclature of this group is still in a state of flux, we opt here to simply refer our specimens to P.cf.punctatus, until the ongoing revision of the group is complete.

####### Genus *Rhoptropus* Peters, 1869

##### ﻿ Rhoptropus aff. barnardi

Taxon classificationAnimaliaSquamataGekkonidae

Hewitt, 1926

193A95A0-55E5-5A3A-8597-A771FECA37F7

Fig. 6h

###### Records.

N’Dolondolo [-13.8133, 13.1316, 681 m] (MUNHAC/MB03-001592− 001595); vic. N’Dolondolo [-13.8113, 13.1365, 699 m] (MUNHAC/ MB03-001596−001603); Basecamp 1 [-13.7770, 13.2591, 1488 m] (MUNHAC/MB03-001604−001610); Maylowe [-13.8355, 13.2755, 798 m] (CAS 266105–10, 266130–32, 266134–37, 266156; MUNHAC/MB03-001611−001620); Catchi, rock outcrops near basecamp [-13.7653, 13.2571, 1645 m] (CAS 266127; MUNHAC/MB03-001621, 001622); near Basecamp 1 [-13.7770, 13.2591, 1488 m] (MUNHAC/ MB03-001623−001633; CAS 266164–68); Catchi surroundings [-13.7619, 13.2568, 1585 m] (MUNHAC/MB03-001634−001688); Catchi, rock outcrops near basecamp [-13.7653, 13.2571, 1645 m] (MUNHAC/MB03-001689−001693); MPLA post near Catchi [-13.7618, 13.2514, 1614 m] (MUNHAC/MB03-001694); 2 km N of Maylowe [-13.8289, 13.2625, 820 m] (MB03-001695−001705).

###### Comments.

Recent surveys in southwestern Angola revealed undescribed cryptic diversity associated with *Rhoptropusbarnardi* (Ceríaco et al. 2016; Kuhn 2016; Butler et al. 2019; Lobón-Rovira et al. 2022). The specimens collected in Serra da Neve belong to an undescribed Angolan endemic with affinities to *Rhoptropusbarnardi* Hewitt, 1926 and *R.biporosus* FitzSimons, 1957, which is widespread in southwestern Angola, from sea level to an elevation of more than 2000 m. A revision of the genus is being prepared (DP unpubl. data).

##### ﻿ Rhoptropus aff. montanus

Taxon classificationAnimaliaSquamataGekkonidae

Laurent, 1964

B9641044-C3EC-5D0D-A4D0-AEE5249296FE

Fig. 7a

###### Records.

Catchi surroundings [-13.7618, 13.2514, 1614 m] (MUNHAC/MB03-001706−001708).

**Figure 7.**
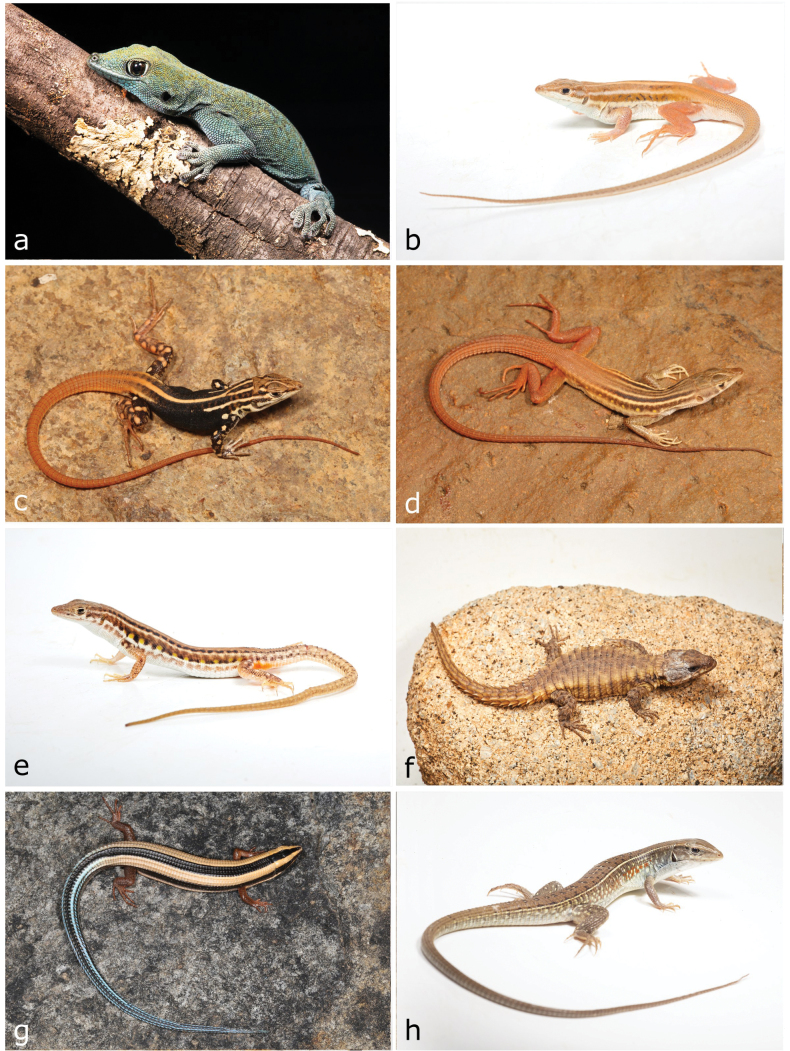
**a**Rhoptropusaff.montanus**b***Helioboluscrawfordi* (adult) **c***Helioboluscrawfordi* (juvenile) **d***Pedioplanishaackei***e***Pedioplanisserodioi***f***Cordylusphonolithos***g***Cordylosaurussubtessellatus***h***Gerrhosaurus* sp. Photographs by AT (**a, b, e, f, h**) and LMPC (**c, d, g**).

###### Comments.

Three specimens of an unknown species of *Rhoptropus* were collected in a riparian area during our last expedition in 2022. This lineage is currently being described in a separate paper, and is endemic to the highlands of the inselberg (DP unpubl. data). It has affinities to *Rhoptropusmontanus* Laurent, 1964, another montane endemic from the Huila Plateau in southwestern Angola (Laurent 1964).

#### ﻿Family Lacertidae Oppel, 1811

##### Genus *Heliobolus* Fitzinger, 1843

###### ﻿ Heliobolus crawfordi

Taxon classificationAnimaliaSquamataLacertidae

Marques, Ceríaco, Heinicke, Chehouri, Conradie, Tolley & Bauer, 2022

605094AE-8A51-56E2-AD24-3466B30DC237

Fig. 7b, c

####### Records.

N’Dolondolo [-13.8133, 13.1316, 681 m] (CAS 266267–266269, 266271, 266273, 266275; INBAC/AMB 10335); near Maylowe [-13.8113, 13.3222, 879 m] (CAS 266120, 266121, 266146; INBAC/LMPC 1220, 1221; MHNC-UP/REP 869–871); 2 km N of Maylowe [-13.8280, 13.2416, 804 m] (MUNHCA/MB03-001709−001713).

####### Comments.

Endemic to Angola and restricted to the central coastal regions of the country, including the surroundings of Serra da Neve (Marques et al. 2022). It is absent from higher elevations in Serra da Neve but abundant in the surrounding lowlands.

##### Genus *Pedioplanis* Fitzinger, 1843

###### ﻿ Pedioplanis haackei

Taxon classificationAnimaliaSquamataLacertidae

Conradie, Measey, Branch & Tolley, 2012

66C7EE55-8C8A-5DA5-832D-BCE922DBCBE1

Fig. 7d

####### Records.

Dirt road to the top of the mountain, near Maylowe [-13.8328, 13.2652, 794 m] (MHNC-UP/REP 634-635, CAS 266123); Dirt road to Quilengues [-13.8159, 13.3264, 892 m] (MHNC-UP/REP 645); vic. N’Dolondolo [-13.8086, 13.13521, 731 m] (CAS 264757, 264765); 2 km N of Maylowe [-13.8280, 13.2625, 820 m] (MUNHAC/MB03-001714−001716).

####### Comments.

This species is endemic mainly to Namibe Province, usually occurring near granite outcrops on sandy areas (Conradie et al. 2012; Parrinha et al. 2021). It was only found in the lowland arid plains of Serra da Neve surroundings, in Mopane habitats, but not in the Miombo-dominated areas at higher altitudes.

###### ﻿ Pedioplanis serodioi

Taxon classificationAnimaliaSquamataLacertidae

Parrinha, Marques, Heinicke, Khalid, Parker, Tolley, Childers, Conradie, Bauer & Ceríaco, 2021

096689AF-E072-5E1D-9B7F-FCA13587A378

Fig. 7e

####### Records.

Dirt road to the top of the mountain, near Maylowe [-13.8328, 13.2652, 794 m] (MHNC-UP/REP 636; CAS 266122); Dirt road to Quilengues [-13.8159, 13.3264, 892 m] (MHNC-UP/REP 637, 638); Maylowe [-13.8355, 13.2755, 798 m] (MHNC-UP/REP 639, 646); 2 km N of Maylowe [-13.8280, 13.2625, 820 m] (MUNHAC/MB03-001717−001756).

####### Comments.

This recently described species (Parrinha et al. 2021) is widely distributed through the lowlands of southwestern Angola, from central Benguela Province to western Cunene Province, with exception of the more xeric areas of southwestern Namibe Province. As with other lacertids, this species was only recorded from the arid lowlands of the inselberg.

#### ﻿Family Cordylidae Fitzinger, 1826

##### Genus *Cordylus* Laurenti, 1768

###### ﻿ Cordylus phonolithos

Taxon classificationAnimaliaSquamataCordylidae

Marques, Ceríaco, Stanley, Bandeira, Agarwal & Bauer, 2019

959D1402-FB5F-53B1-9E80-0E019E44C8EC

Fig. 7f

####### Records.

vic. N’Dolondolo [-13.8068, 13.1351, 752 m] (CAS 263581; INBAC/AMB 10272); Rock outcrops near Catchi [-13.7653, 13.2571, 1645 m] (MUNHAC/MB03-001757−001765).

####### Comments.

This recently described species is only known from the Serra da Neve inselberg and is considered a strict endemic. *Cordylusphonolithos* was recently described from the inselberg by Marques et al. (2019). It is genetically divergent and morphologically distinguished from the closely related Angolan Escarpment dwelling *Cordylusmachadoi* Laurent, 1964 and the low-elevation species *C.namakuiyus* Stanley, Ceríaco, Bandeira, Valério, Bates & Branch, 2016. This species is found in cracks in granite boulders, but sometimes can be seen basking outside or even crossing paths on the ground.

#### ﻿Family Gerrhosauridae Fitzinger, 1843

##### Genus *Cordylosaurus* Gray, 1866

###### ﻿ Cordylosaurus subtessellatus

Taxon classificationAnimaliaSquamataGerrhosauridae

(Smith, 1844)

62C465C4-F9E6-5CBD-8708-81EBF66B451F

Fig. 7g

####### Records.

vic. Dolondolo [-13.8087, 13.1352, 731 m] (CAS 263031); Mamué riparian area [-13.8003, 13.1229, 710 m] (INBAC/AMB 10326).

####### Comments.

The species is known from southwestern Angola through western Namibia and into western parts of South Africa (Marques et al. 2018). In Angola, *C.subtessellatus* has been recorded from the coastal areas of Benguela and Namibe provinces (Marques et al. 2018). It is commonly found basking in granite outcrops and hidden in crevices.

##### Genus *Gerrhosaurus* Wiegmann, 1828

###### ﻿ Gerrhosaurus

Taxon classificationAnimaliaSquamataGerrhosauridae

sp.

DE957F23-FD2F-5379-BFBD-0856B2E5F2E9

Fig. 7h

####### Records.

Catchi surroundings [-13.7577, 13.2543, 1576 m] (MUNHAC/MB03-001766−001778).

####### Comments.

The Serra da Neve population belongs to a genetic clade that also occurs in the plateau areas of central and southeastern Angola, already signaled by Butler et al. (2019) and Conradie et al. (2016b, 2022) (LMPC unpub. data).

##### Genus *Matobosaurus* Bates & Tolley, 2013

###### ﻿ Matobosaurus maltzahni

Taxon classificationAnimaliaSquamataGerrhosauridae

(De Grys, 1938)

4AFD24DB-3CB6-55A1-8FA3-5B7BB598D5CD

Fig. 8a

####### Records.

N’Dolondolo [-13.8133, 13.1362, 681 m] (INBAC/AMB 10280); Rock outcrops near Catchi [-13.7653, 13.2571, 1645 m] (MUNHAC/MB03-001779); 2 km N of Maylowe [-13.8280, 13.2625, 820 m] (MUNHAC/MB03-001780).

**Figure 8.**
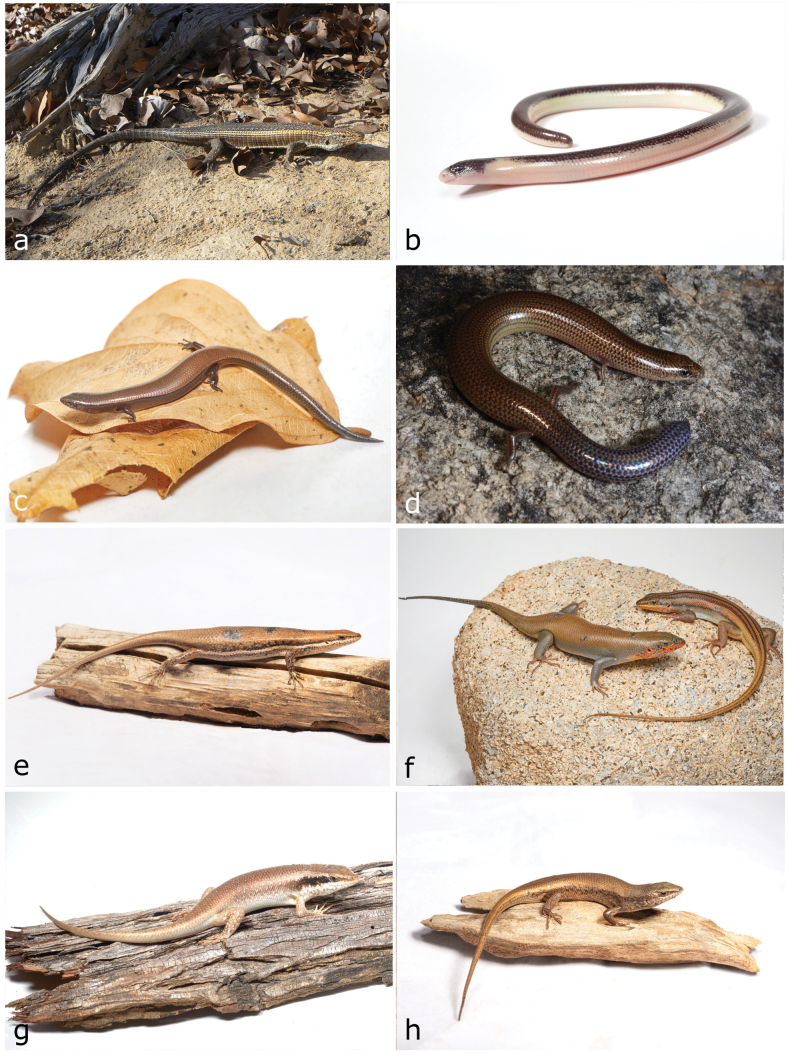
**a***Matobosaurusmaltzahni***b***Acontiasmukwando***c***Panaspis* sp. 1 **d***Sepsinacopei***e***Trachylepisalbopunctata***f***Trachylepisansorgii***g***Trachylepisbinotata***h***Trachylepisbouri*. Photographs by AT (**a–c, e–h**); LMPC (**d**).

####### Comments.

The species is known from the northwestern regions of Namibia to the southwestern regions of Angola, where it occurs in the western lowlands of Namibe and Benguela provinces and neighboring Huíla Province (Marques et al. 2018). It occurs in a variety of micro-habitats, from rock outcrops to woodlands. The specimen MUNHAC/MB03-001780 was found at night, sleeping inside a hollow trunk of a Mopane tree.

#### ﻿Family Scincidae

##### Genus *Acontias* Cuvier, 1816 “1817”

###### ﻿ Acontias mukwando

Taxon classificationAnimaliaSquamataScincidae

Marques, Parrinha, Tiutenko, Lopes-Lima, Bauer & Ceríaco, 2023

BCEE8BC5-6C4C-54D4-A767-9807A07F115B

Fig. 8b

####### Records.

Catchi, Miombo woodland near basecamp [-13.7660, 13.2587, 1674 m] (MUHNAC/MB03-001522–24).

####### Comments.

This recently described species is only known from the Serra da Neve inselberg and considered a strict endemic. It was found hiding under rocks and active on leaf-litter in Miombo-dominated landscapes (Marques et al. 2023b).

##### Genus *Mochlus* Günther, 1864

###### ﻿ Mochlus sundevallii

Taxon classificationAnimaliaSquamataScincidae

(Smith, 1849)

90867A7E-A683-5F6D-9978-A8908044B0F0

####### Record.

2 km N of Maylowe [-13.8280, 13.2625, 820 m] (specimen not collected).

####### Comments.

This species occurs throughout southern Africa (Marques et al. 2018). The specimen was seen active at dusk, under a cattle fence made from dead Mopane trees, in sandy soil with some Mopane leaf litter.

##### Genus *Panaspis* Cope, 1868

###### ﻿ Panaspis cabindae

Taxon classificationAnimaliaSquamataScincidae

(Bocage, 1866)

3F807B56-0DC7-5611-A66A-FC5FEDDFCAE1

####### Records.

Mamué riparian area [-13. 8015, 13.1206, 665 m] (CAS 263550, 263553–263555; UF 187242; INBAC/AMB 10317, 10320).

####### Comments.

The distribution area of this species extends from central Africa to the central highlands in Angola, reaching its southern limit in the forest margins below the Escarpment in southeastern Namibe Province (Ceríaco et al. 2020b). The species appears to be absent from more xeric and desertic areas of the southwestern regions of Namibe Province, where it is replaced by its congener *Panaspismocamedensis*. In Serra da Neve, the species was found under leaf-litter, in dense Miombo woodlands. In Serra da Neve the species occurs in more humid lowlands, tendentially preferring more forested areas with deeper leaf litter.

###### ﻿ Panaspis mocamedensis

Taxon classificationAnimaliaSquamataScincidae

Ceríaco, Heinicke, Parker, Marques & Bauer, 2020

372C0D1A-D824-5733-A845-25564F49A56F

####### Records.

2 km N of Maylowe [-13.8280, 13.2625, 820 m] (MB03-001532, 001533).

####### Comments.

*P.mocamedensis* is endemic to Namibe Province (Ceríaco et al. 2020b). This species tendentially prefer more open and dry micro-habitats than their Angolan congeners.

###### ﻿ Panaspis

Taxon classificationAnimaliaSquamataScincidae

sp. 1

91691C79-3B5A-5207-8C49-E858F2437BA5

Fig. 8c

####### Records.

Rock outcrops near Catchi [-13.7653, 13.2571, 1645 m] (MUNHAC/MB03-001525, 001526); Catchi, basecamp [-13.7627, 13.2562, 1597 m] (MUNHAC/MB03-001528); 2 km N of Maylowe [-13.8280, 13.2625, 820 m], (MUNHAC/MB03-001529–001531); Dry riverbed, 2 km N of Maylowe [-13.8265, 13.2601, 720 m] (MUNHAC/MB03-001534).

####### Comments.

A new species is currently being described (MPM unpubl. data), only known from the Serra da Neve inselberg, where it is assumed to be endemic. *Panaspis* sp. 1 belongs to the same Central African lineages as *P.cabindae* and is part of the same clade as the Gulf of Guinea Oceanic islands species (Ceríaco et al. 2020b; MPM unpubl. data). This species shows some ecological adaptability occurring in both Miombo woodlands in the higher elevation areas but also in the arid Mopane lowlands (MPM unpubl. data). It was recorded in sympatry with *P.mocamedensis* in the lowlands of the inselberg.

###### ﻿ Panaspis

Taxon classificationAnimaliaSquamataScincidae

sp. 2

CDF38553-201F-5372-A1EC-DE3FF3222ED8

####### Record.

MPLA post near Catchi [-13.7618, 13.2514, 1614 m] (MUNHAC/MB03-1527).

####### Comments.

A new species is currently being described (MPM unpubl. data); similarly to *Panaspis* sp. 1, it is known only from Serra da Neve. The single collected specimen was found under a long near to a riparian gallery. *Panaspis* sp. 2 present phylogenetic and biogeographic affinities with *P.annettesabinae* from the highlands of Ethiopia (Colston et al. 2020; MPM unpubl. data).

##### Genus *Sepsina* Bocage, 1866

###### ﻿﻿ Sepsina copei

Taxon classificationAnimaliaSquamataScincidae

Bocage, 1873

17B218A6-66F5-5D0D-82C5-2297745387AC

Fig. 8d

####### Records.

Mamué riparian area [-13.8015, 13.1206, 665 m] (CAS 263918–263921); vic. N'Dolondolo [-13.8086, 13.1352, 731 m] (CAS 263916).

####### Comments.

This species is endemic to Angola. It occurs in western coastal regions, from Luanda to Namibe Province.

##### Genus *Trachylepis* Fitzinger, 1843

###### ﻿ Trachylepis albopunctata

Taxon classificationAnimaliaSquamataScincidae

(Bocage, 1867)

3D089568-5654-5C51-A443-218C68C1B822

Fig. 8e

####### Records.

Rock outcrop near Basecamp 1 [-13.7864, 13.2575, 1596 m] (CAS 263560); Basecamp 1 [-13.7770, 13.2591, 1488 m] (MUNHAC/MB03-001384, 001516; INBAC/LMPC 1265); Catchi surroundings [-13.7620, 13.2569, 1585 m] (MUNHAC/MB03-001468, 001469, 001486–001498, 001500–001502, 001504–001508); 2 km N of Maylowe [-13.8280, 13.2625, 818 m] (MUHNAC/MB03-001509); Catchi, rock outcrops near basecamp [-13.7653, 13.2571, 1645 m] (MUNHAC/MB03-001499, 001503).

####### Comments.

The species is widely distributed in Angola, except for the southeastern areas of the country, where it is replaced by *Trachylepisdamarana* (Peters, 1870). The Angolan population was long identified as *Trachylepisvaria* (Peters, 1867), but recent reviews by Weinell and Bauer (2018) and Ceríaco et al. (2024) validated the specific status of *albopunctata*.

###### ﻿ Trachylepis ansorgii

Taxon classificationAnimaliaSquamataScincidae

(Boulenger, 1907)

E69E698E-369D-52E5-A2EF-73062F8C80B2

Fig. 8f

####### Records.

Maylowe [-13.8357, 13.2763, 800 m] (MUHNAC/MB03-001396, 001397); Basecamp 1 [-13.7770, 13.2591, 1488 m] (CAS 263567; UF 187313); Rock outcrop near Basecamp 1 [-13.7865, 13.2572, 1594 m] (UF 187314); vic. N’Dolondolo [-13.8104, 13.1361, 713 m] (CAS 263545); Catchi, rock outcrops near basecamp [-13.7653, 13.2571, 1645 m] (MUNHAC/MB03-001472, 001475, 001477, 001478, 001482); Catchi surroundings [-13.7620, 13.2569, 1585 m] (MUNHAC/MB03-001473, 001474, 001479–001481, 001485); 2 km N of Maylowe [-13.8280, 13.2625, 818 m] (MUHNAC/MB03-001476); Lutala crater, near airstrip [-13.7325, 13.1841, 1567 m] (MUHNAC/MB03-1484).

####### Comments.

Butler et al. (2019) presented the first topotypical material since the taxon was originally described by Boulenger (1907). According to the molecular results of Butler et al. (in press) and Ceríaco et al. (2024), *T.ansorgii* is a valid species, distinct from *T.sulcata*. *Trachylepisansorgii* is restricted to the highlands of Angola, mostly distributed from Malanje to northern Huíla Province, while *T.sulcata* seems to occur in the southwestern regions of the country, with records for southern Namibe and southwestern Huíla provinces (Ceríaco et al. 2024). The material collected from Serra da Neve is the first evidence of *T.ansorgii* in Namibe Province and the southernmost record of the species, emphasizing the species restriction to areas of high elevations.

###### ﻿ Trachylepis binotata

Taxon classificationAnimaliaSquamataScincidae

(Bocage, 1867)

5E3D3C05-3A41-5A5D-846B-93A991CAAA89

Fig. 8g

####### Records.

Maylowe [-13.8357, 13.2763, 800 m] (CAS 266149); 2 km N of Maylowe [-13.8280, 13.2625, 818 m] (MUHNAC/MB03-001453, 001454).

####### Comments.

This large arboreal skink occurs across all southwestern Angola. *Trachylepisbinotata* is usually associated with Mopane woodland habitats and was widely recorded in the country (Marques et al. 2018; Ceríaco et al. 2024). In our study area, it was only found in the Mopane-dominated localities at the base of the inselberg.

###### ﻿ Trachylepis bouri

Taxon classificationAnimaliaSquamataScincidae

Ceríaco, Marques, Parrinha, Tiutenko, Weinell, Butler & Bauer, 2024

7409C5D7-3904-589C-90FB-2073AE799B7E

Fig. 8h

####### Records.

Catchi surroundings [-13.7620, 13.2569, 1585 m] (MUNHAC/MB03-001511); Catchi, rock outcrops near basecamp [-13.7653, 13.2571, 1645 m] (MUNHAC/MB03-001512).

####### Comments.

This newly described species is endemic to southwestern Angola. It is associated with rock outcrops, especially along the Escarpment (Ceríaco et al. 2024).

###### ﻿ Trachylepis chimbana

Taxon classificationAnimaliaSquamataScincidae

(Boulenger, 1887)

D7609D9B-A7C2-55B2-8C3C-C4EC55494F7F

Fig. 9a

####### Records.

vic. N’Dolondolo [-13.8105, 13.1361, 707 m] (CAS 263542, 263543, 263544); near dirt road to top of the mountain, N of Maylowe [-13.8105, 13.2581, 1502 m] (CAS 263562, 263563); Maylowe [-13.8355, 13.2755, 798 m] (MUNHAC/MB03-001387); 2 km N of Maylowe [-13.8280, 13.2625, 818 m] (MUHNAC/MB03-001518); Catchi surroundings [-13.7619, 13.2569, 1585 m] (MUNHAC/MB03-001514); Catchi, rock outcrops near basecamp [-13.7653, 13.2571, 1645 m] (MUNHAC/MB03-001515, 001519).

**Figure 9.**
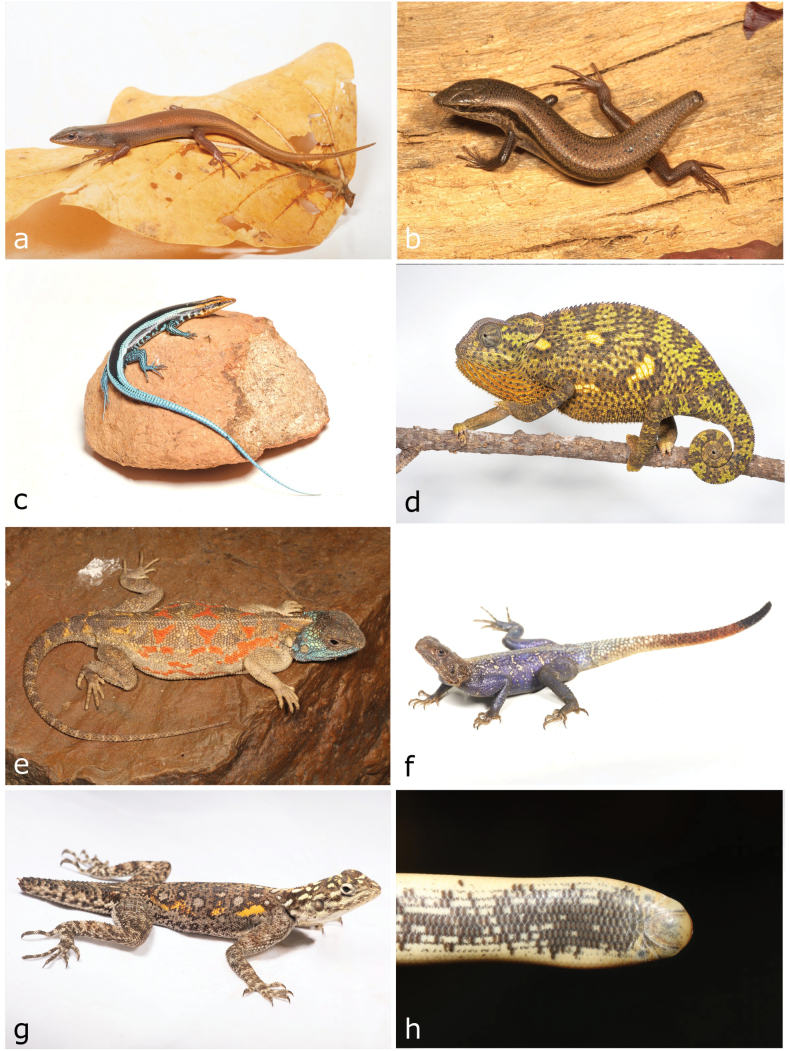
**a***Trachylepischimbana***b***Trachylepishuilensis***c***Trachylepislaevis***d***Chamaeleodilepis***e***Agamaaculeata***f***Agamaschacki* (male) **g***Agamaschacki* (female) **h***Afrotyphlopsschlegelipetersii*. Photographs by AT (**a, c, d, f, g**) and LMPC (**b, e, h**).

####### Comments.

*Trachylepischimbana* is one of the most taxonomically challenging species of the genus. This Angolan endemic had been confused by several authors (Schmidt 1933; Hellmich 1957a, b; Laurent 1964) with other taxa, such as *Trachylepisbocagii* or *T.wahlbergi*. Ceríaco et al. (2024) restricted *T.chimbana* to northern Namibe and southern Benguela provinces. This species is primarily rupicolous and was found mainly on granite outcrops.

###### ﻿ Trachylepis huilensis

Taxon classificationAnimaliaSquamataScincidae

(Laurent, 1964)

1368B37D-9DA8-5B8B-AF3A-E1EEC33872B5

Fig. 9b

####### Records.

Basecamp 1 [-13.7770, 13.2591, 1488 m] (CAS 263565; UF 187310; MUNHAC/MB03-001388, 001516); Rock outcrop near Basecamp 1 [-13.7877, 13.2572, 1600 m] (UF 187311), [-13.7865, 13.2572, 1594 m] (CAS 263558), [-13.7881, 13.2571, 1612 m] (CAS 263559); Catchi surroundings [-13.7619, 13.2569, 1585 m] (MUNHAC/MB03-001467, 001470, 001471, 001517).

####### Comments.

This taxon was originally described as a subspecies of *T.bayonii* by Laurent (1964). Butler et al. (2019) provided the first evidence that *huilensis* should be considered a full species, rather than a subspecies. Based on newly obtained molecular and morphological data, Ceríaco et al. (2024) found that the species is more widespread than originally known. It is associated with the highlands of the Leba Escarpment in Huíla Province and the Serra da Neve inselberg.

###### ﻿ Trachylepis laevis

Taxon classificationAnimaliaSquamataScincidae

(Boulenger, 1907)

30185359-538A-56BB-9045-A36B20FE7D71

Fig. 9c

####### Records.

vic. N’Dolondolo [-13.8105, 13.1361, 713 m] (CAS 263541; UF 187308); Rock outcrop near Basecamp 1 [-13.7881, 13.2571, 1612 m] (CAS 263582); vic. Catci, granite boulders near basecamp [-13.7646, 13.2601, 1603 m] (MUHNAC/MB03-001463–001465).

####### Comments.

This conspicuous species is common in southwestern Angola and neighboring Namibia (Marques et al. 2018; Ceríaco et al. 2024). It is usually seen basking on granite outcrops.

#### ﻿Family Chamaeleonidae Rafinesque, 1815

##### Genus *Chamaeleo* Linnaeus, 1758

###### ﻿ Chamaeleo dilepis

Taxon classificationAnimaliaSquamataChamaeleonidae

Leach, 1819

BB04EDC0-6F7A-5959-9038-DF0375782D22

Fig. 9d

####### Records.

Catchi surroundings [-13.7620, 13.2569, 1585 m] (MUNHAC/MB03-001781−001782).

####### Comments.

*Chamaeleodilepis* is a species complex distributed throughout southern and eastern Africa (Tilbury 2010). Marques et al. (2018) opted to identify all Angolan records as *C.dilepisquilensis* and showed its large distribution throughout the country. More recently, Main et al. (2022) provided evidence of several divergent lineages that may warrant a species-level status. For this paper and until the taxonomy of the group is clarified, we simply refer to the Serra da Neve populations as *C.dilepis*.

#### ﻿Family Agamidae Gray, 1827

##### Genus *Agama* Daudin, 1802

###### ﻿ Agama aculeata

Taxon classificationAnimaliaSquamataAgamidae

Merrem, 1820

5569665E-93D2-529F-9925-B8F3E3E343EC

Fig. 9e

####### Records.

Maylowe [-13.8342, 13.2767, 803 m] (CAS 266138, 266139).

####### Comments.

*Agamaaculeata* is a ground-dwelling agamid commonly found in higher altitudes in southern Angola (Marques et al. 2018). In Namibe Province the species is restricted to areas closely associated with inselbergs and with the Great Escarpment, being replaced by *A.anchietae* in lower altitudes (Marques et al. 2018).

###### ﻿ Agama schacki

Taxon classificationAnimaliaSquamataAgamidae

Mertens, 1938

35EFA3CC-075E-5A7F-8653-CFF00B9A60C2

Fig. 9f, g

####### Records.

Rock outcrop near Basecamp 1 [-13.7865, 13.2572, 1594 m] (CAS 263035), [-13.7881, 13.2571, 1612 m] (INBAC/AMB 10233); N’Dolondolo [-13.8133, 13.1362, 681 m] (INBAC/AMB 10302, 10303); vic. N’Dolondolo [-18.8105, 13.1361, 707 m] (CAS 263026); Maylowe [-13.83424, 13.27669, 803 m] (CAS 266128, CAS 266129; INBAC/LMPC 1169; MUNHAC/MB03-001783); Catchi surroundings [-13.7620, 13.2569, 1584 m] (MUNHAC/MB03-001784); Catchi, rock outcrops near basecamp [-13.7653, 13.2571, 1645 m] (MUNHAC/MB03-001785–001796); 2 km N of Maylowe [-13.8280, 13.2616, 804 m] (MUNHAC/MB03-001797); Serra da Neve base, 2 km N of Maylowe, rock outcrops near basecamp [-13.8280, 13.2625, 820 m] (MUNHAC/MB03-001798).

####### Comments.

Originally described by Mertens (1938) as a subspecies of *Agamaplaniceps* Peters, 1862, *A.schacki* was shown to be a distinct species by Butler et al. (in press), based on molecular data. Marques et al. (2018) had already considered *A.schacki* as a full species and restricted its distribution to the Escarpment area in western Huíla and eastern Namibe and Benguela provinces. The species is strongly associated with higher elevations, being replaced by *A.planiceps* in lowland areas of southern Namibe Province.

#### ﻿Family Typhlopidae

##### Genus *Afrotyphlops* Broadley & Wallach, 2009

###### ﻿ Afrotyphlops schlegeli petersii

Taxon classificationAnimaliaSquamataTyphlopidae

(Bocage, 1873)

70D5BB68-75F4-517A-9ABF-2FF546D2053B

Fig. 9h

####### Record.

Mamué riparian area [-13.8015, 13.1206, 665 m] (CAS 266467).

####### Comments.

The *Afrotyphlopsschlegeli* species complex is, as most of the other members of this genus, a taxonomic and nomenclatural conundrum. The validity of *petersii* as a distinct taxon, endemic to southwestern Angola and Namibia, was supported by Roux-Estève (1974). Marques et al. (2018) considered the Angolan population simply as *A.schlegeli*. Given the significant geographic separation between the Angolan population and the topotypical population in Mozambique, as well as the morphological differences noted by Roux- Estève (1974), we treat the Angolan populations as a distinct subspecies until a more comprehensive review of the group is undertaken. This taxon appears to be associated with higher elevation and montane areas (Marques et al. 2018). The collected specimen had been killed by locals who regard it as highly venomous.

#### ﻿Family Leptotyphlopidae

##### Genus *Leptotyphlops* Fitzinger, 1843

###### ﻿ Leptotyphlops cf. scutifrons

Taxon classificationAnimaliaSquamataLeptotyphlopidae

(Peters, 1854)

A0083551-F411-5EC5-84E6-59D08DC2ADCF

####### Record.

vic. Mamué [-13.7877, 13.1257, 1600 m] (CAS 264756).

####### Comments.

*Leptotyphlopsscutifrons* is widely distributed in southern Africa. It was recorded from several localities in the Angolan Plateau (Broadley and Broadley 1999; Conradie et al. 2016b; Marques et al. 2018). Although some historical records may refer to *Namibianalatifrons* (Sternfeld, 1908) (Marques et al. 2018), our specimen agrees almost completely with the diagnosis of *L.scutifrons* that was provided by Broadley and Broadley (1999). Nevertheless, this taxon should be treated as a species complex with multiple divergent lineages (Adalsteinsson et al. 2009). In Angola, it is mostly restricted to the highlands of the Escarpment and Central Plateau (Marques et al. 2018).

#### ﻿Family Pythonidae Fitzinger, 1826

##### Genus *Python* Daudin, 1803

###### ﻿ Python natalensis

Taxon classificationAnimaliaSquamataPythonidae

Smith, 1833

4FCE32C7-F5E1-5E48-9A23-98B6C84702AF

Fig. 10a

####### Record.

Maylowe [-13.8342, 13.2767, 803 m] (photographic record, specimens not collected).

**Figure 10.**
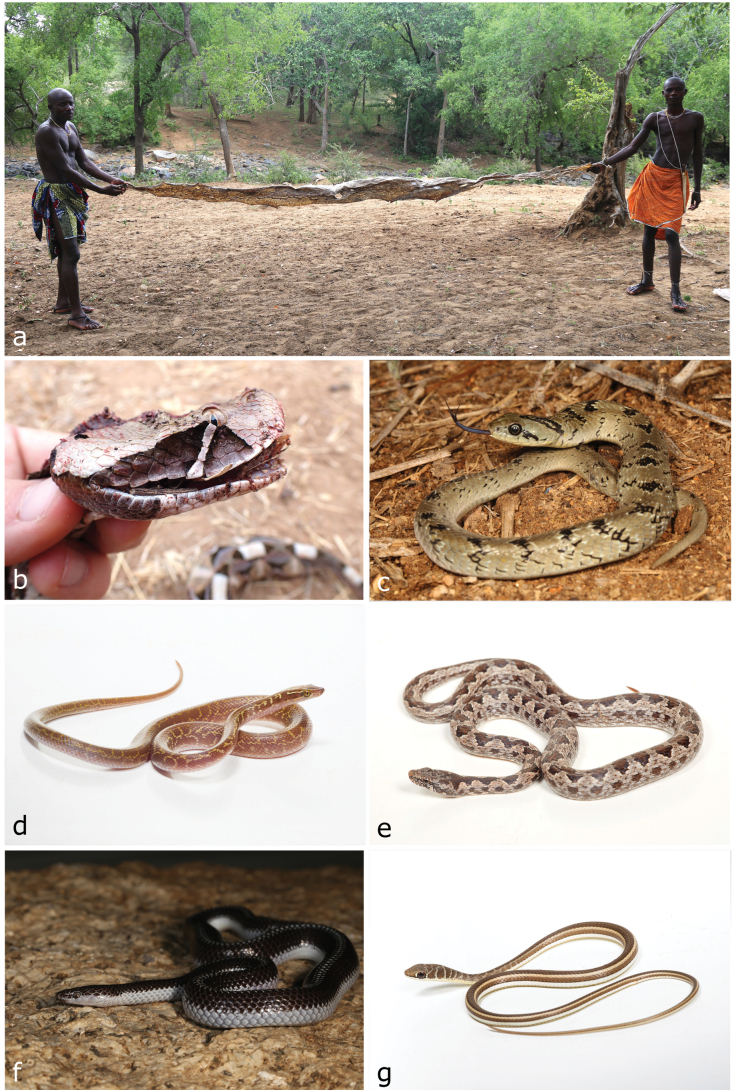
**a***Pythonnatalensis* (skin) **b**Bitis (Macrocerastes) gabonica**c***Caususnasalis***d***Boaedonvariegatus***e***Hemirhagerrhisviperina***f***Lycophidionhellmichi***g***Psammophissubtaeniatus*. Photographs by LMPC (**a**, **c, f**); AT (**d, e, g**) and DP (**b**).

####### Comments.

This species is widely distributed throughout southern Angola (Marques et al. 2018). The skin in the photograph is held by local Mucubal people in the town of Maylowe, where it was killed.

#### ﻿Family Viperidae Oppel, 1811

##### Genus *Bitis* Gray, 1842

**Subgenus Macrocerastes Reuss, 1939**

###### ﻿ Bitis (Macrocerastes) gabonica

Taxon classificationAnimaliaSquamataViperidae

(Duméril, Bibron & Duméril, 1854)

7DFE2383-85F1-5BCF-BB4A-3CD3554C67E7

Fig. 10b

####### Record.

Catchi surroundings [precise locality unknown] (MUNHAC/MB03-001535).

####### Comments.

The Gaboon viper is an African viperid with a primarily west and central African distribution, while some populations extend marginally into northern Zambia. Isolated populations exist from southern South Sudan, Kenya, Tanzania, and the KwaZulu-Natal region of eastern South Africa (Spawls et al. 2023; Marques et al. 2018). In Angola, the species has been recorded mainly in northern and central areas of the country, with the southwestern-most record in Central Benguela (Marques et al. 2018). The Serra da Neve population thus represents the southwestern-most record of the species and the first record for Namibe Province. Our specimen was killed by locals when crossing a path early in the morning. The local name for this species is M’buta, which is also used for other large viperids such as the Puff-adder, *Bitisarietans* Merrem, 1820 (Ceríaco and Marques 2021).

##### Genus *Causus* Wagler, 1830

###### ﻿ Causus nasalis

Taxon classificationAnimaliaSquamataViperidae

Stejneger, 1893

C08DBAD1-AD9D-51E4-9543-DDF1FEF185FD

Fig. 10c

####### Records.

Mamué riparian area [-13.8006, 13.1230, 706 m] (CAS 263034; INBAC/AMB 10324).

####### Comments.

Bocage (1895) suggested that Angolan populations of *Caususresimus* (Peters, 1862) belonged to a new variety, which he named *angolensis*. This decision was supported, according to the author, by morphological differences between the Angolan populations and the nominotypical form. A few years earlier, Stejneger (1893) had already described what he called *Caususnasalis* from “West Africa” based on a specimen collected during the United States Eclipse Expedition to West Africa in 1890. Since Stejneger did not know the exact collection locality of the type, he assumed that it was “Cunga” [most likely Fazenda Cunga, on the banks of the Kwanza River, Luanda Province]. A label bearing this information accompanied another specimen (USNM 16074), which we examined, and which is currently labelled as paratype in the collections of the National Museum of Natural History, Smithsonian Institution, USA (USNM). Neither *nasalis* nor *angolensis* has been commented upon by subsequent authors. The recently collected specimens from Serra da Neve could represent this distinct Angolan population, considering not only its morphological differences but also its geographic isolation from the rest of the known distribution of the topotypical form. Our specimens agree entirely with the morphological description provided by both Stejneger (1893) and Bocage (1895). Given this, we here recognize *C.nasalis* as a valid species for Angola, endemic to the coastal areas of the country, from Luanda to Namibe provinces.

#### ﻿Family Lamprophiidae Fitzinger, 1843

##### Genus *Boaedon* Duméril, Bibron & Duméril. 1854

###### ﻿ Boaedon variegatus

Taxon classificationAnimaliaSquamataLamprophiidae

Bocage, 1867

7C57CED5-6023-51A7-B6B6-E86FE0990E82

Fig. 10d

####### Records.

vic. N’Dolondolo [-13.8105, 13.1361, 707 m] (CAS 263027); Catchi, basecamp [-13.7627, 13.2562, 1597 m] (MUNHAC/MB03-001536).

####### Comments.

This species is a southwestern African endemic, distributed from the coastal areas of Kwanza Sul Province in Angola southwards to Namibia (Hallermann et al. 2020).

##### Genus *Hemirhagerrhis* Boettger, 1896

###### ﻿ Hemirhagerrhis viperina

Taxon classificationAnimaliaSquamataLamprophiidae

(Bocage, 1873)

C3766D52-0326-59C4-991F-BCBFDBD6212F

Fig. 10e

####### Records.

Basecamp 1 [-13.7770, 13.2591, 1488 m] (UF 187211); vic. N’Dolondolo [-13.8105, 13.1361, 291 m] (CAS 263028), [-13.8109, 13.1351, 705 m] (CAS 263032); N’Dolondolo [-13.8133, 13.1362, 681 m] (INBAC/AMB 10279, 10298); Catchi, rock outcrops near basecamp [-13.7653, 13.2571, 1645 m] (MUNHAC/MB03-001537, 001538), [-13.7659, 13.2582, 1671 m] (MUNHAC/MB03-001539); Catchi, basecamp [-13.7627, 13.2562, 1597 m] (MUNHAC/MB03-001540, 001541).

####### Comments.

This species is restricted to southwestern Angola and northern Namibia (Marques et al. 2018). It is relatively common across its range, where it is usually found during the day in rocky outcrops.

##### Genus *Lycophidion* Fitzinger, 1843

###### ﻿ Lycophidion hellmichi

Taxon classificationAnimaliaSquamataLamprophiidae

Laurent, 1964

FFA1275C-DB55-53E2-BAA4-30BD690A8FF6

Fig. 10f

####### Records.

Mauué riparian area [-13.8006, 13.1230, 706 m] (CAS 263033); Catchi surroundings [-13.7620, 13.2569, 1585 m] (MUNHAC/MB03-001542, 001543).

####### Comments.

This recently collected material represents the first Angolan specimens of this species collected since its original description by Laurent (1964). The species appears to be endemic to southwestern Angola, from Benguela to Namibe Province and to northwestern Namibia. Our specimen from Serra da Neve was collected in the middle of its currently known distribution (Marques et al. 2018). A specimen from northwestern Namibia was assigned to this species by Broadley (1991, 1996). A very similar species, *L.namibianum* Broadley, 1991, which occurs in northwestern Namibia and southern Namibe Province (Broadley 1991, 1996; Lobón-Rovira et al. 2022), is distinguished from *L.hellmichi* by the lack of contact of the post-nasal with the first labial (in contact in *L.hellmichi*). Our specimens agree with the holotype in both coloration and the contact between post-nasal and first supralabial (Laurent 1964). The specimens from Catchi are adult male and female collected while copulating.

##### Genus *Psammophis*

###### ﻿ Psammophis subtaeniatus

Taxon classificationAnimaliaSquamataLamprophiidae

Peters, 1882

81F8BE84-99FA-507A-8E05-87D4ED78EE54

Fig. 10g

####### Records.

Maylowe [-13.8342, 13.2767, 803 m] (CAS 266140); Catchi surroundings [-13.7620, 13.2569, 1585 m] (MUNHAC/MB03-001544); 2 km N of Maylowe [-13.8280, 13.2625, 803 m] (MUNHAC/MB03-001545).

####### Comments.

Species restricted to dry shrublands and savannas, particularly Mopane woodland, and widely distributed from southern Angola and northern Namibia eastwards through Botswana, Zambia and Zimbabwe to western Mozambique (Marques et al. 2018). Specimen MUNHAC/MB03-001545 was collected on sandy substrate near a rocky outcrop while active at night.

##### Genus *Psammophylax* Fitzinger, 1843

###### ﻿ Psammophylax tritaeniatus

Taxon classificationAnimaliaSquamataLamprophiidae

(Günther, 1868)

DCC84890-3629-51BB-83FE-9935F7FE9FA5

Fig. 11a

####### Record.

Catchi surroundings [-13.7620, 13.2569, 1585 m] (MUNHAC/MB03-001546).

**Figure 11.**
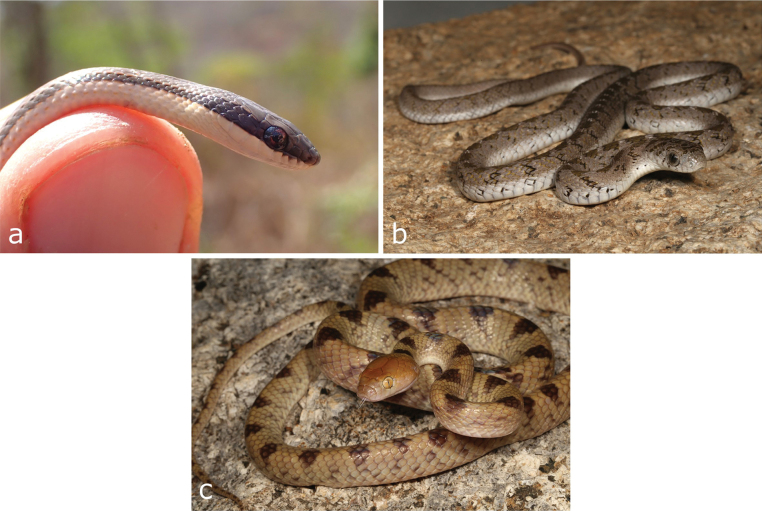
**a***Psammophylaxtritaeniatus***b***Dasypeltisscabra***c***Telescopussemiannulatuspolystictus*. Photographs by DP (**a**) and LMPC (**b, c**).

####### Comments.

This species occurs in mid to high-elevation areas throughout southern and eastern Africa (Spawls et al. 2018). Previous records from Angola are from high-elevation areas of the Escarpment (Marques et al. 2018). This specimen represents the first record for Namibe Province and the westernmost record for *P.tritaeniatus*. Further sampling across the species range may reveal additional cryptic diversity (Keates et al. 2019).

#### ﻿Family Colubridae

##### Genus *Dasypeltis* Wagler, 1830

###### ﻿ Dasypeltis scabra

Taxon classificationAnimaliaSquamataColubridae

(Linnaeus, 1758)

72EC8C9F-CAA6-5E1D-BB36-6CAB2EE1B2AA

Fig. 11b

####### Record.

N’Dolondolo [-13.8133, 13.1362, 681 m] (CAS 263023).

####### Comments.

Following the key provided by Bates (2023), our specimen completely conforms with *Dasypeltisscabra*. Marques et al. (2018) provided a map of the distribution of this widespread species in Angola.

##### Genus *Telescopus* Wagler, 1830

###### ﻿ Telescopus semiannulatus polystictus

Taxon classificationAnimaliaSquamataColubridae

Mertens, 1954

81B8F7CD-6985-5505-AC8C-9B8CFBB22C87

Fig. 11c

####### Records.

vic. N’Dolondolo [-13.8105, 13.1361, 707 m] (CAS 263029); 2 km N of Maylowe [-13.8280, 13.2625, 803 m] (MUNHAC/MB03-001547).

####### Comments.

The collected specimens exhibit more than 60 dark blotches on the back. This fits in the range attributed to the subspecies polystictus rather than to the nominotypical form (20–50 blotches according to Branch 1998). This is the first confirmed record for Angola of this subspecies that is otherwise known from neighboring Namibia.

## ﻿Discussion

Serra da Neve is situated in one of the most herpetologically rich areas not only in Angola, but in Southwest Africa more broadly (Marques et al. 2018). Due to its close proximity to the high elevation areas of the Angolan Escarpment, in combination with the surrounding lowland xeric areas of Namibe and Benguela provinces, the inselberg combines two distinct faunas: one associated with the Angolan highlands (with some Zambezian or Congolian affinities), and one with the desert areas extending from the Namib desert in Namibia to Benguela Province in Angola.

Despite its small area, the number of species recorded from the Serra da Neve inselberg is high, especially when strictly endemic species are considered. The number of Serra da Neve endemics is almost the same as in other biodiversity hotspots in the region, such as the Angolan Central Plateau and the Huíla Escarpment, whose areas are many times the size of the inselberg. These numbers are also considerably higher than those found on other similar inselbergs in Namibia. Whether these differences are related to the geomorphological and biogeographic characteristics of Serra da Neve, isolation, or simply reflect a bias due to poor sampling in other inselbergs, remains to be tested. Despite the already high number of recorded herpetological taxa for Serra da Neve and surrounding areas, several other taxa are expected to occur in the area, given their habitat preferences and known distribution ranges (Marques et al. 2018). Although amphibian diversity is lower in more arid than in mesic areas, several other xeric-tolerant species, such as *Poyntonophrynusdombensis* (Bocage, 1895) and *Sclerophrysregularis* (Reuss, 1833), are expected to occur in Serra da Neve and nearby lowlands. The lack of records of members of the family Hyperoliidae, such as representatives of the *Hyperoliusangolensis* Ahl, 1931 or *H.benguellensis*/*nasutus* species complexes, may be because none of our sampling events occurred in the peak of the rainy season. The habitats in the higher areas of the inselberg are similar to those where these species are found in other parts of the country. Other common rocket-frogs, such as *Ptychadenaoxyrhynchus* (Smith, 1849) may also be expected in the study area. The same applies to certain squamate groups, such as representatives of the lacertid genera *Ichnotropis* and *Nucras*, amphisbaenids, rock monitors (*Varanusalbigularisangolensis* Schmidt, 1933), or tree agamas of the genus *Acanthocercus*. To our surprise, despite the relatively high diversity of geckos in the area, we did not record the ubiquitous *Hemidactylusmabouia* (Moreau de Jonnès, 1818), a species commonly found around human settlements. This may be another sign of the isolation from routes of regular human movements on and around the inselberg. Other species of geckos, such as the recently described *Kolekanosspinicaudus* Lobón-Rovira, Conradie, Baptista & Pinto, 2022, *Rhoptropusbenguellensis* Mertens, 1938, and Pachydactyluscf.oreophilus McLachlan & Spence, 1967 may be present in the northern surrounding areas of the inselberg. Snake records always tend to be limited, especially during such short-term surveys and, therefore, many other species are expected to occur in the area, such as *Amblyodipsaspolylepis* (Bocage, 1873), *Aparallactuscapensis* Smith, 1849, *Atractaspisbibronii* Smith, 1849, *Dispholidustypus* (Smith, 1828), *Prosymna* sp., *Philothamnus* sp., *Najaanchietae* Bocage, 1879, and *N.nigricincta* Bogert, 1940 in both the lowland and highland areas; *Bitisarietans*, *B.caudalis* (Smith, 1839), and *Aspidelapslubricus* (Laurenti, 1768) in the lowlands; and *Crotaphopeltishotamboeia* (Laurenti, 1768), *Dendroaspispolylepis* Günther, 1864, *Elapsoidea* sp., and *Thelotorniscapensisoatesi* (Günther, 1881) in the highlands. The discovery of other unexpected and undescribed taxa cannot be ruled out, as seen from the recent descriptions of such species as *Acontiasmukwando* and *Lygodactylusbaptistai*.

The remoteness of Serra da Neve and the lack of good accesses to its base and summit have served so far as a guarantee of its preservation. Furthermore, the ruggedness, elevation, and climatic conditions of the inselberg may deter human settlement and concomitant large-scale habitat alterations. In oceanic islands, topographic and climatic conditions are known to have prevented land-cover changes, despite the presence of humans (Norder et al. 2020). This seems to apply to inselbergs such as Serra da Neve as well. A quick comparison with Mount Moco, an inselberg of similar elevation and with similar number of human inhabitants seems to confirm this idea: Mount Moco is much less rugged than Serra da Neve, and much easier access by the local population has led to a considerable land-use change (Powell et al. 2023). Due to its remoteness, Serra da Neve is unlikely to suffer from activities such as mining, that traditionally cause degradation of inselbergs (Porembski et al. 2016).

Landscape changes are clearly visible on the different plateaus of Serra da Neve, especially around human settlements with large agricultural fields and cattle pastures. Human-caused fires were observed during our fieldwork. The presence of livestock, such as cows, goats, and pigs were also noticeable, but no rodent species associated with humans, such as rats, were observed or collected in an ongoing small mammals survey. Traditional hunting is common, targeting ungulates and birds, but not reptiles. However, chameleons and snakes are seen as dangerous and are usually killed when encountered, as it was the case of our specimens of *Bitisgabonica*, *Telescopussemiannulatuspolystictus*, *Psammophylaxtritaeniatus*, and *Lycophidionhellmichi*. At the base of the inselberg, tortoises, such as *Kinixys*, are consumed or traded as a delicacy. The remaining amphibians and reptiles are usually neglected by the local human population. Pollution is noticeable in a few water bodies due to its use by humans and cattle and may pose a threat to some amphibians.

Serra da Neve was identified as a potential conservation area by both the scientific community (Huntley and Matos 1992; Huntley 2010; Pinto et al. 2023) and the Angolan authorities. The present checklist, even though it is focused only on amphibians and reptiles, unambiguously shows its conservation importance and interest, not only due to its high number of endemic taxa, but also to its taxonomic diversity. Similar checklists are currently being prepared for other taxonomic groups, such as birds and mammals (Marks pers. comm. November 2022; Ferguson pers. comm. November 2022). This is particularly important in the current scenario of climate change, as inselbergs in xeric areas can serve as important biodiversity retreats (Burke 2003).

## Supplementary Material

XML Treatment for Xenopus petersii

XML Treatment for Poyntonophrynus grandisonae

XML Treatment for Poyntonophrynus pachnodes

XML Treatment for Sclerophrys pusilla

XML Treatment for Phrynomantis annectens

XML Treatment for Leptopelis anchietae

XML Treatment for Ptychadena anchietae

XML Treatment for Phrynobatrachus natalensis

XML Treatment for Amietia angolensis

XML Treatment for Tomopterna ahli

XML Treatment for Tomopterna tuberculosa

XML Treatment for Kinixys belliana

XML Treatment for Stigmochelys pardalis

XML Treatment for Afroedura praedicta

XML Treatment for Hemidactylus benguellensis

XML Treatment for Chondrodactylus pulitzerae

XML Treatment for Lygodactylus baptistai

XML Treatment for Lygodactylus nyaneka

XML Treatment for Pachydactylus caraculicus

XML Treatment for Pachydactylus maiatoi

XML Treatment for Pachydactylus cf. punctatus

XML Treatment for Rhoptropus aff. barnardi

XML Treatment for Rhoptropus aff. montanus

XML Treatment for Heliobolus crawfordi

XML Treatment for Pedioplanis haackei

XML Treatment for Pedioplanis serodioi

XML Treatment for Cordylus phonolithos

XML Treatment for Cordylosaurus subtessellatus

XML Treatment for Gerrhosaurus

XML Treatment for Matobosaurus maltzahni

XML Treatment for Acontias mukwando

XML Treatment for Mochlus sundevallii

XML Treatment for Panaspis cabindae

XML Treatment for Panaspis mocamedensis

XML Treatment for Panaspis

XML Treatment for Panaspis

XML Treatment for Sepsina copei

XML Treatment for Trachylepis albopunctata

XML Treatment for Trachylepis ansorgii

XML Treatment for Trachylepis binotata

XML Treatment for Trachylepis bouri

XML Treatment for Trachylepis chimbana

XML Treatment for Trachylepis huilensis

XML Treatment for Trachylepis laevis

XML Treatment for Chamaeleo dilepis

XML Treatment for Agama aculeata

XML Treatment for Agama schacki

XML Treatment for Afrotyphlops schlegeli petersii

XML Treatment for Leptotyphlops cf. scutifrons

XML Treatment for Python natalensis

XML Treatment for Bitis (Macrocerastes) gabonica

XML Treatment for Causus nasalis

XML Treatment for Boaedon variegatus

XML Treatment for Hemirhagerrhis viperina

XML Treatment for Lycophidion hellmichi

XML Treatment for Psammophis subtaeniatus

XML Treatment for Psammophylax tritaeniatus

XML Treatment for Dasypeltis scabra

XML Treatment for Telescopus semiannulatus polystictus

## ﻿Additional information

### Author ORCIDs

Mariana P. Marques https://orcid.org/0000-0002-1712-2632

Diogo Parrinha https://orcid.org/0000-0002-1302-025X

Manuel Lopes-Lima https://orcid.org/0000-0002-2761-7962

Arthur Tiutenko https://orcid.org/0000-0001-5952-4923

Aaron M. Bauer https://orcid.org/0000-0001-6839-8025

Luis M. P. Ceríaco https://orcid.org/0000-0002-0591-9978

### Data availability

All of the data that support the findings of this study are available in the main text.
